# ﻿New species of *Eupolyphaga* Chopard, 1929 and *Pseudoeupolyphaga* Qiu & Che, 2024 (Blattodea, Corydioidea, Corydiinae), with notes on their female genitalia

**DOI:** 10.3897/zookeys.1211.128805

**Published:** 2024-09-04

**Authors:** Wei Han, Yan-Li Che, Pei-Jun Zhang, Zong-Qing Wang

**Affiliations:** 1 College of Plant Protection, Southwest University, Chongqing 400715, China Southwest University Chongqing China; 2 Key Laboratory of Agricultural Biosafety and Green Production of Upper Yangtze River (Ministry of Education), Southwest University, Chongqing 400715, China Southwest University Chongqing China

**Keywords:** Cockroach, Dictyoptera, Polyphagini, spermatheca

## Abstract

Two new species of *Eupolyphaga* (*E.bicolor* Han, Che & Wang, **sp. nov.** and *E.nigra* Han, Che & Wang, **sp. nov.**) and six new species of *Pseudoeupolyphaga* (*P.flava* Han, Che & Wang, **sp. nov.**, *P.deficiens* Han, Che & Wang, **sp. nov.**, *P.magna* Han, Che & Wang, **sp. nov.**, *P.longiseta* Han, Che & Wang, **sp. nov.**, *P.latizona* Han, Che & Wang, **sp. nov.**, and *P.baimaensis* Han, Che & Wang, **sp. nov.**) are described and illustrated. The female external genitalia and spermathecae of these two genera are reported and the role of these characters in species delimitation is discussed.

## ﻿Introduction

*Eupolyphaga* was once the most diverse genus of Corydioidea in China, containing 22 species and four subspecies (Han et al. 2022). Combined with morphological characteristics and phylogenetic reconstruction results, Han et al. (2024) revised *Eupolyphaga* and transferred most of the species to *Pseudoeupolyphaga* Qiu & Che, 2024. Therefore, only seven species are now included in *Eupolyphaga*, and 15 species and four subspecies are included in *Pseudoeupolyphaga*.

For a long time, the species identification of the two genera was mainly based on some male external morphology (body color, size, maculae distribution in tegmina) and the shape of ootheca serrations (Chopard 1922, 1929; Feng and Woo 1988; Woo and Feng 1992; Qiu et al. 2018; Han et al. 2022). Female characters, such as spermatheca, basivalvula, and spermathecal plate can help to distinguish between *Pseudoeupolyphaga* from *Eupolyphaga* (Han et al. 2024). To determine whether these female characters could be used for species identification, more samples are needed to evaluate their reliability. In addition to morphological characterization, molecular data have also been used for species delimitation in the two genera and have proven to be effective and appropriate (Han et al. 2022).

Following a recent collection, we found that some specimens collected from Yunnan and Sichuan provinces showed high morphological resemblance to some of the known *Pseudoeupolyphaga* species, while also presenting subtle differences. For example, a male specimen from Guanyinqiao Township of Sichuan exhibited external morphology similar to *P.yunnanensis* (Chopard, 1922), although the former is notably larger in body size. Similarly, some male specimens from Tazigou closely resembled *P.simila* (Qiu, 2022), but the former has significantly shorter tegmina and hind wings. Whether these differences are interspecific or intraspecific variation also needed to be clarified.

Therefore, in this study, we combined morphological characters and molecular data to delimit species of *Eupolyphaga* and *Pseudoeupolyphaga*. We describe two new species of *Eupolyphaga* and six new species of *Pseudoeupolyphaga*, provide illustrations of female genitalia and spermathecae, and discuss the taxonomic significance of these female characters. This helps to explore the diversity of *Eupolyphaga* and *Pseudoeupolyphaga* and provides a basis for identifying females of these two genera.

## ﻿Materials and methods

### ﻿Material

All specimens studied in this article are deposited in College of Plant Protection, Southwest University, Chongqing, China (**SWU**). The terminology used in this article mainly follows Roth (2003) (external morphology), Klass (1997) (male genitalia) and McKittrick (1964) (female genitalia). The formulation “median sclerites” follows Qiu et al. (2018). The terminal three or four segments of the abdomen were excised and immersed in a 10% NaOH solution and heated for 30 min to eliminate fat. Subsequent procedures, including morphological dissection of males, DNA extraction, and PCR and sequencing, adhere to the methodology outlined by Han et al. (2022).

### ﻿Sequence processing and phylogenetic analyses

A total of 42 sequences were analyzed, comprising 40 in-group and 2 out-group sequences [*Eucorydiadasytoides* (Walker, 1868) and *Diplopterapunctata* (Eschscholtz, 1822)], as detailed in Table 1. All 21 newly acquired sequences have been submitted to GenBank (https://www.ncbi.nlm.nih.gov/nuccore) with accession numbers PQ059675 to PQ059695. Alignment of all COI fragments was performed using the MUSCLE algorithm within MEGA 11 (Kumar et al. 2016), ensuring translatability into protein sequences. Genetic distances, both interspecific and intraspecific, were computed employing the Kimura 2-parameter model (Kimura 1980). PartitionFinder v. 2.1.1 (Lanfear et al. 2017) was utilized to determine the optimal partitioning scheme and substitution models with default parameters (COI_pos 1: SYM+I+G; COI_pos 2: GTR+I+G; COI_pos 3: GTR+G). Maximum-likelihood analysis involved ten independent likelihood searches, selecting the highest likelihood result. Node and branch supports were assessed via IQ-TREE v. 2.2.0 (Nguyen et al. 2015) employing 10,000 ultrafast bootstrap (UFBoot) replicates. Additionally, the “-bnni” option was employed to mitigate severe model violations.

**Table 1.** Samples used in species delimitation.

Species	Abbreviation	GenBank ID	Collecting information	Remark
*P.baimaensis* sp. nov.	PseuBaim	PQ059685	Baima Village, Sichuan; 4 Aug. 2019; Lu Qiu	male
*P.latizona* sp. nov.	PseuLatiSM	PQ059683	Caoke Village, Sichuan; 20 Jul. 2022; Wei Han, Xin-Xing Luo	female
	PseuLatiDB1	PQ059691	Danba County, Sichuan; 12 Jul. 2017; Jian-Yue Qiu, Hao Xu	male
	PseuLatiDB2	PQ059692	Jiaju Zangzhai, Sichuan; 12 Jul. 2017; Jian-Yue Qiu, Hao Xu	male
*P.longiseta* sp. nov.	PseuLong1	PQ059684	Baima Snow Mountain, Sichuan; 27 Jul. 2020; Wei Han, Xin-Xing Luo, Lin Guo	female
	PseuLong2	PQ059677	Baima Snow Mountain, Sichuan; 27 Jul. 2020; Wei Han, Xin-Xing Luo, Lin Guo	nymph
*P.flava* sp. nov.	PseuFlav	PQ059689	Liude Village, Yunnan; 9 Jul. 2021; Lu Qiu, Hao Xu	female
*P.magna* sp. nov.	PseuMagn	PQ059688	Jinchuan County, Sichuan; 2020; Jian-Yue Qiu	male
*P.deficiens* sp. nov.	PseuDefiHS	PQ059687	Heishui County, Sichuan; 22 Jun. 2021; Lu Qiu, Hao Xu	nymph
	PseuDefiCJS	PQ059686	Cuoji Mountain, Sichuan; 6 Aug. 2019; Lu Qiu	female
*P.fusca*	PseuFusc1	PQ059678	Cang Mountain, Yunnan; 29 Jul. 2022; Wei Han, Xin-Xing Luo	nymph
	PseuFusc2	PQ059680	Cang Mountain, Yunnan; 29 Jul. 2022; Wei Han, Xin-Xing Luo	male
*P.pilosa*	PseuPiloLDT	PQ059681	Luodatang countryside, Yunnan; 25 Jul. 2022; Wei Han, Xin-Xing Luo, Lin Guo	female
	PseuPiloWBS	PQ059690	Wenbi Mountain, Yunnan; 24 Jul. 2022; Wei Han, Lin Guo	male
	PseuPiloYL	PQ059682	Lanyue Valley, Yunnan; 24 Jul. 2022; Wei Han, Lin Guo	male
	PseuPiloWX	OP215882	/	/
*P.fengifengi*	PseuFengZXS	PQ059693	Zixi Mountain, Yunnan; 31 Jul. 2022; Wei Han, Xin-Xing Luo	female
	PseuFengDHS	PQ059679	Dahei Mountain, Sichuan; 22 Jul. 2022; Wei Han, Xin-Xing Luo	female
	PseuFeng1	OP215870	/	/
	PseuFeng2	OP215871	/	/
*P.simila*	PseuSimiMYL	OP215883	/	/
	PseuSimiDGC	PQ059676	Dagou Village, Li County, Sichuan; 22 Apr. 2023; Wei Han	male
	PseuSimiTZG	PQ059675	Tazigou, Parktou Township, Li County, Sichuan; 18 Apr. 2023; Wei Han	male
*P.dongi*	PseuDong	OP215872	/	/
*P.nigrinotum*	PseuNigr	OP215879	/	/
*P.wooi*	PseuWooi	OP215874	/	/
*P.daweishana*	PseuDawe	OP215877	/	/
*P.yunnanensis*	PseuYunnTM	OP215869	/	/
	PseuYunnCY	OP215865	/	/
	PseuYunnBM	OP215866	/	/
*P.reducta*	PseuRedu	OP215886	/	/
*P.xuorum*	PseuXuor	OP215875	/	/
*E.sinensis*	EupoSine	OP215846	/	/
*E.hanae*	EupoHana	OP215849	/	/
*E.hupingensis*	EupoHupi	OP215854	/	/
*E.robusta*	EupoRobu	OP215856	/	/
*E.miracidia*	EupoMira	OP215878	/	/
*E.udenostyla*	EupoUden	OP215887	/	/
*E.bicolor* sp. nov.	EupoBico	PQ059694	Guiling, Guangxi; 14 Feb. 2023; Hao-Fei Fan	male
*E.nigra* sp. nov.	EupoNigr	PQ059695	Zhubu Village, Guangxi; 7 Jul. 2023; Wei Han, Xin-Ran Li	male
**Outgroup**				
*Eucorydiadasytoides*	EucoDasy	LC480880	/	/
*Diplopterapunctata*	DiplPunc	MF479156	/	/

## ﻿Results

### ﻿Molecular analysis based on COI

The alignment of the 42 COI sequences encompasses a total of 660 nucleotide sites, with intra- and interspecific distances detailed in Suppl. material 3. The interspecific genetic distances between species in *Eupolyphaga* range from 9.8% (between *E.hanae* Qiu, Che & Wang, 2018 and *E.robusta* Qiu, Che & Wang, 2018) to 20.86% (between *E.sinensis* (Walker, 1868) and *E.nigra* sp. nov.). In *Pseudoeupolyphaga*, we found similar interspecific variations, with the largest interspecific distance recorded at 20.90% between *P.yunnanensis* and *P.latizona* sp. nov., and the smallest at 6.61% between *P.pilosa* (Qiu, Che & Wang, 2018) and *P.fusca* (Chopard, 1929). In terms of intraspecific genetic distance, a maximum of 7.54% was observed between samples from Wenbi Mountain and Luodatang countryside of *P.pilosa*.

**Figure 1.**
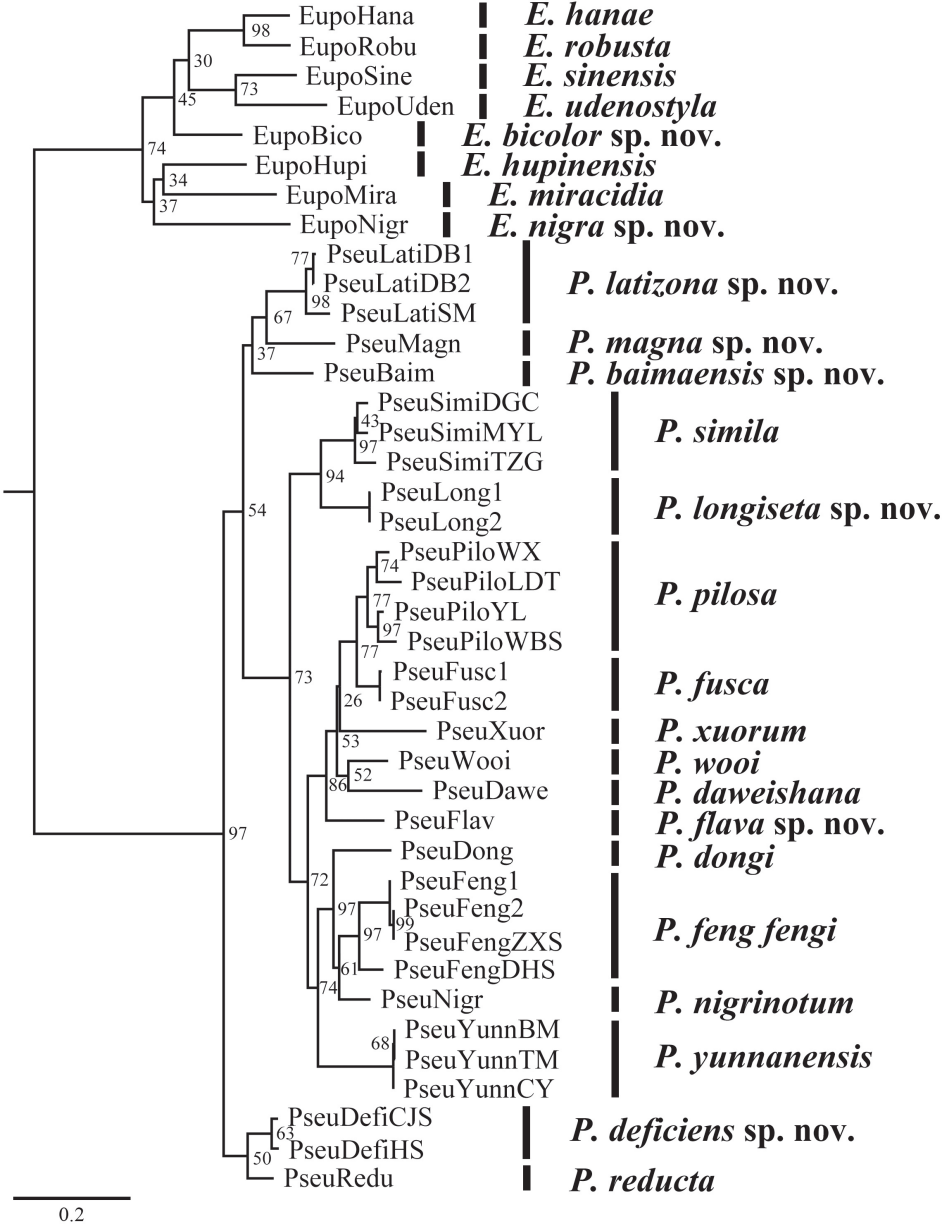
Phylogenetic tree of *Eupolyphaga* and *Pseudoeupolyphaga* inferred by maximum-likelihood (ML) analysis of the mitochondrial COI fragment (outgroups not shown). UFBoot values are shown at the nodes.

The phylogenetic tree of *Eupolyphaga* and *Pseudoeupolyphaga*, derived from the COI sequence, is depicted in Fig. 1. The maximum likelihood (ML) tree illustrates the monophyletic nature of species distinguished by morphology, although almost all the branches exhibit low support values and a few species were represented by a single terminal, so their respective monophyly was not tested.

### ﻿Taxonomy

#### ﻿Genus Eupolyphaga

Taxon classificationAnimaliaBlattodeaCorydiidae

Chopard, 1929

9C691BA3-DEE4-5390-949E-EA0F5D0496D1

Eupolyphaga Chopard, 1929: 261; Bey-Bienko 1950: 283; Princis 1962: 53; Feng et al. 1997: 165; Qiu et al. 2018: 5; Han et al. 2024: 165.

##### Type species.

*Polyphagasinensis* Walker, 1868, by original designation.

##### Supplementary diagnosis.

The external structure and male genitalia characteristics have been given and discussed in Qiu et al. (2018) and Han et al. (2024). So only female characteristics are added below: Supra-anal plate (TX) distinctly pubescent, with a slightly protruded posterior margin. Paraprocts (pp.) pubescent, the inner side extending to the middle in a curved hook. The two median sclerites generally wedged. Cerci short, not exceeding the posterior margin of supra-anal plate, setose and pubescent. Paratergites (pt.) irregularly-banded. Crosspiece (cp.) nearly transparent, with a small protuberance pointing toward the posterior lobes of valvifer II (sp.pl.). The base of posterior lobes of valvifer II fused with the anterior arch (aa.), forming into a circinate structure. Posterior lobes of valvifer II curved, the terminal part generally rounded. First valvule (v.I) long, basally connected to the basivalvula (bsv.) and spermathecal plate, gradually tapering from the base to the tip. Basal part of valvifer II (v.II) and valvifer III (v.III) enlargement apparent, apex part sharp. Basivalvula symmetrical, with generally flat anterior margins, curly lateral margin, and round posterior edge. Spermathecal plate well-sclerotized, with the middle of the trailing edge folding backwards, two lobes symmetrical. Spermatheca (sp.) consists of ampulla and spermathecal duct. The ampulla mostly globular, and the spermathecal duct usually bifurcated. Vestibular sclerite (vst.s) shaped like the letter “W”, with protrusions on both sides and in the middle. Subgenital plate (SVII) densely setose, posterior margin protruded and the terminal part emarginate medially.

#### ﻿ Eupolyphaga bicolor

Taxon classificationAnimaliaBlattodeaCorydiidae

Han, Che & Wang sp. nov.

5841A660-9A86-58BB-AF43-BA47E5C55D5A

https://zoobank.org/34FF402F-9BF4-4C3C-8002-9D0AB626DC39

**Figure 2.**
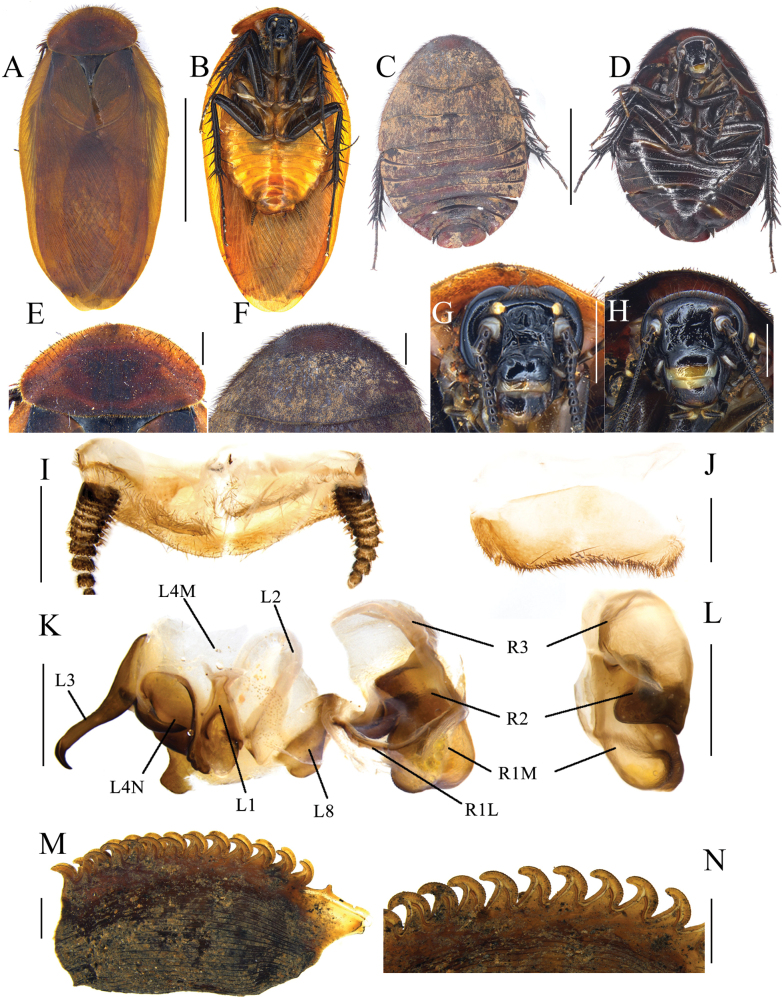
*Eupolyphagabicolor* Han, Che & Wang, sp. nov. **A** male holotype, dorsal view **B** male holotype, ventral view **C** female paratype, dorsal view **D** female paratype, ventral view **E** male pronotum, dorsal view **F** female pronotum, dorsal view **G** male head, ventral view **H** female head, ventral view **I** supra-anal plate, ventral view **J** subgenital plate, ventral view **K** genitalia, dorsal view **L** right phallomere, right-ventral view **M** ootheca, lateral view **N** ootheca, close-up view to show the serration. Scale bars: 1.0 cm (**A–D**); 0.2 cm (**E–L**); 0.1 cm (**M, N**).

Figs 2A–N , 4A, B , 5A, I

##### Type material.

***Holotype***: China • male; Guangxi Zhuang Autonomous Region, Guiling City; 14 Feb. 2023; Hao-Fei Fan leg. ***Paratype***: China • 1 female, same collection data as holotype.

##### Diagnosis.

This species is smaller in male size compared to other congeneric species (body length 16.7–23.4 mm) except *E.miracidia* (12.1–12.5 mm). It resembles *E.sinensis* and *E.hanae* by its yellow abdomen, but it can be distinguished by its almost unicolored tegmina as well as black head and legs (Fig. 2A, B, G). In addition, the serrations on the keel of this species are distinctly more curved than those of *E.hanae*, and approximate those of *E.sinensis* and *E.miracidia*.

##### Description.

**Male holotype. *Measurements* (mm).** Overall length (including tegmen): 23.51; body length: 15.96; body width (tegmina not included): 8.14; tegmen length × width: 19.66 × 7.77; pronotum length × width: 7.20 × 3.59.

***Coloration*.** Head and face black. Ocelli pale yellowish. Antennal sockets white. Antenna blackish brown. Ante-clypeus whitish and subtransparent (Fig. 2G). Pronotum and tegmina yellowish brown. Hind wings pale brown. Legs black, coxa and trochanter slightly yellowish brown. Pulvilli and arolia white. Abdomen bright yellow (Fig. 2A, B).

***Body*. *Head***: Sub-rounded, hidden under the pronotum. Interocular space narrower than the distance between ocelli, and the latter narrower than the distance between antennal sockets. Ocelli distinct, ocelli ridge slightly curved, with a row of setae on the upper edge. Clypeus developed (Fig. 2G). ***Pronotum***: Transverse oval, widest point near the middle. Anterior whitish margin indistinct. Surface covered with long setae (Fig. 2E). ***Tegmina and hind wings***: Nearly unicolored, extending beyond the end of abdomen (Fig. 2A, B). ***Legs***: Slender, front femur type C_1_. Pulvilli and arolia present (Fig. 2B). ***Abdomen***: Supra-anal plate transverse, pubescent, posterior margin protruded medially. Paraprocts simple. Cerci short. Subgenital plate densely setose along the lateral and posterior margins, the hind margin slightly concave in the middle. Styli small and short (Fig. 2I, J). ***Genitalia***: Basal part of L1 prolonged, two hind lobes robust. L2 curved. Genital hook (L3) long, the hooked part curved. Right phallomere long. R1M expanded. R1L banded. R2 simple, the basis chunk rounded and the distal flat. R3 broad and concave (Fig. 2K, L).

**Male paratype.** Similar to the holotype, only legs slightly paler in color.

**Female paratype.** Body length: 22.25; body width: 15.25; pronotum length × width: 10.93 × 5.31.

***Coloration*.** Terga reddish brown. Sterna dark reddish brown. Face black. Ocelli and ante-clypeus yellow. Antennal sockets white. The distal part of labrum pale yellow. Legs black, spines reddish black (Fig. 2C, D, F, H).

The widest point of the pronotum near the hind margin (Fig. 2F). Ocelli distinct, the interocular space larger than the distance between antennal sockets, and larger than the distance between the ocelli (Fig. 2H). Arolia and pulvilli absent. Posterior margin of the supra-anal plate (TX) protruded and emarginated medially. Cerci short, not exceeding the posterior margin of supra-anal plate. Paraprocts (pp.) pubescent, curved hook-like extensions long and robust. The two median sclerites present (Fig. 4A). Paratergites (pt.) banded, irregularly shaped. Crosspiece (cp.) weakly sclerotized, the protrusion long. Apex of posterior lobes of valvifer II (p.l.) slightly curved. Spermathecal plate (sp.pl.) narrow, concave in the middle, the two lobes each having an arch in the middle (Fig. 5A). The ampulla of spermatheca (sp.) large and spherical. The middle part and left part of spermathecal duct expanded. The right bifurcated duct expands and bifurcates again in the center, one of the bifurcated ducts connected to a small globular enlargement, while the other is curved and attached to several expansions (Fig. 5I). Basivalvula (bsv.) transverse, two lobes wide, anterior margin flat, lateral margin curly (Fig. 5A). Vestibular sclerite (vst.s.) shaped like a “W”, with widened apices on both sides and a forked tip in the middle. Subgenital plate (SVII) densely setose, posterior margin protruded, slightly concave in the middle (Fig. 4B).

**Nymph.** Unknown.

**Ootheca.** Yellowish brown. The longitudinal lines distinct. Serrations on the keel large and curved. The space between the serrations of the curved portion distinct. Respiratory canals well developed (Fig. 1M, N).

##### Natural history.

Found in the dry soil beside a cave entrance (Hao-Fei Fan pers. comm., Feb. 2023).

##### Etymology.

The species epithet is derived from the Latin word *bicolor*, which indicates that males of this species have two distinct colors: blackish head and legs; yellowish tegmina, hind wings and abdomen.

#### ﻿ Eupolyphaga nigra

Taxon classificationAnimaliaBlattodeaCorydiidae

Han, Che & Wang sp. nov.

50F8290A-9A91-5F0D-992F-3733506E04B3

https://zoobank.org/FE3E5490-7D2A-480C-9A25-A2A445DCADFC

**Figure 3.**
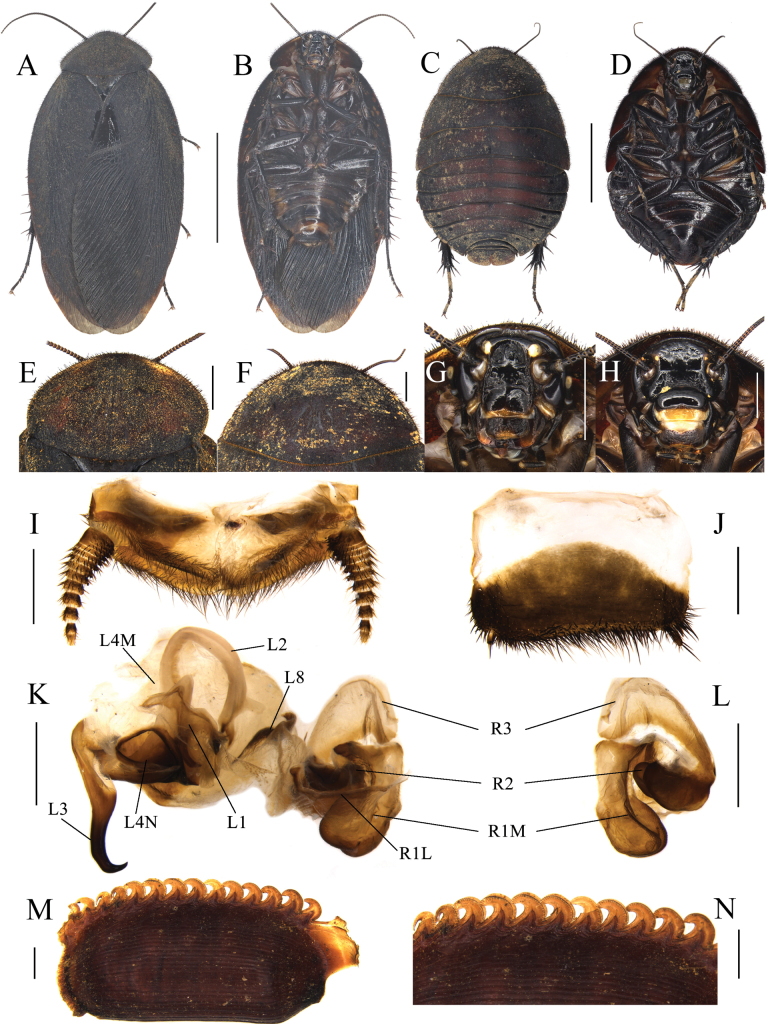
*Eupolyphaganigra* Han, Che & Wang, sp. nov. **A** male holotype, dorsal view **B** male holotype, ventral view **C** female paratype, dorsal view **D** female paratype, ventral view **E** male pronotum, dorsal view **F** female pronotum, dorsal view **G** male head, ventral view **H** female head, ventral view **I** supra-anal plate, ventral view **J** subgenital plate, ventral view **K** genitalia, dorsal view **L** right phallomere, right-ventral view **M** ootheca, lateral view **N** ootheca, close-up view to show the serration. Scale bars: 1.0 cm (**A–D**); 0.2 cm (**E–L**); 0.1 cm (**M, N**).

**Figure 4.**
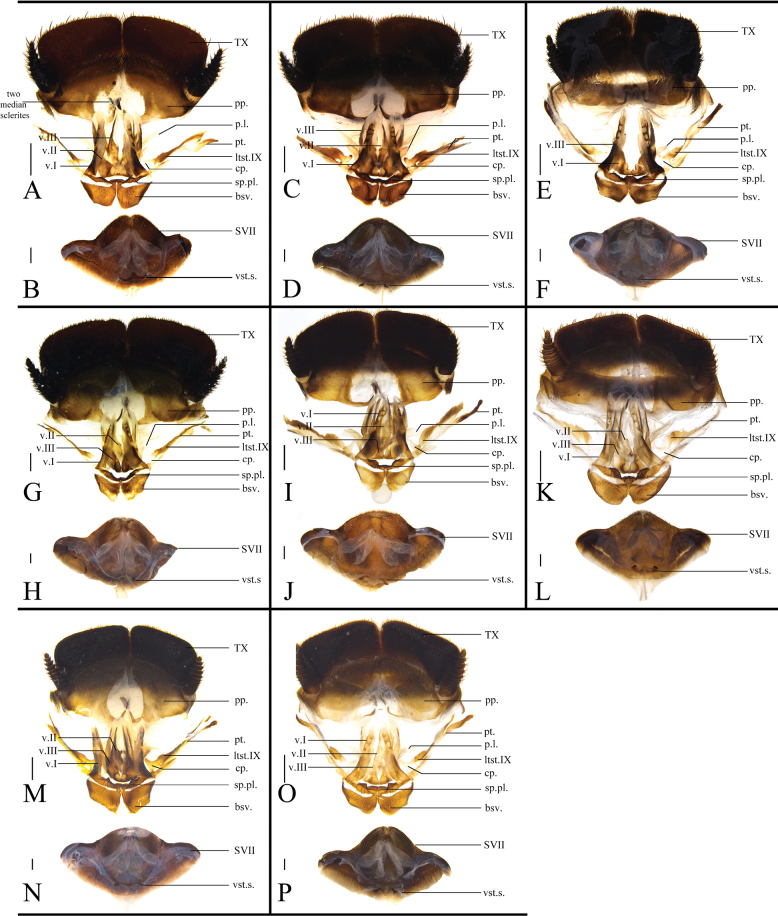
Female external genitalia of eight *Eupolyphaga* species (supra-anal plate and subgenital plate included) **A, B***E.bicolor* Han, Che & Wang, sp. nov. **C, D***E.nigra* Han, Che & Wang, sp. nov. **E, F***E.udenostyla* Qiu, 2022 **G, H***E.hupingensis* Qiu, Che & Wang, 2018 **I, J***E.miracidia* Qiu, 2022 **K, L***E.sinensis* (Walker, 1868) **M, N***E.robusta* Qiu, Che & Wang, 2018 **O, P***E.hanae* Qiu, Che & Wang, 2018. Abbreviations: bsv. basivalvula, cp. crosspiece, ltst. IX laterosternite IX, p.l. posterior lobes of valvifer II, pp. paraprocts, pt. paratergites, sp.pl. spermathecal plate, SVII subgenital plate, TX supra-anal plate, v.I first valvule, v.II second valve, v.III third valve, vst.s vestibular sclerite. Scale bars: 0.1 cm (**A–P**).

**Figure 5.**
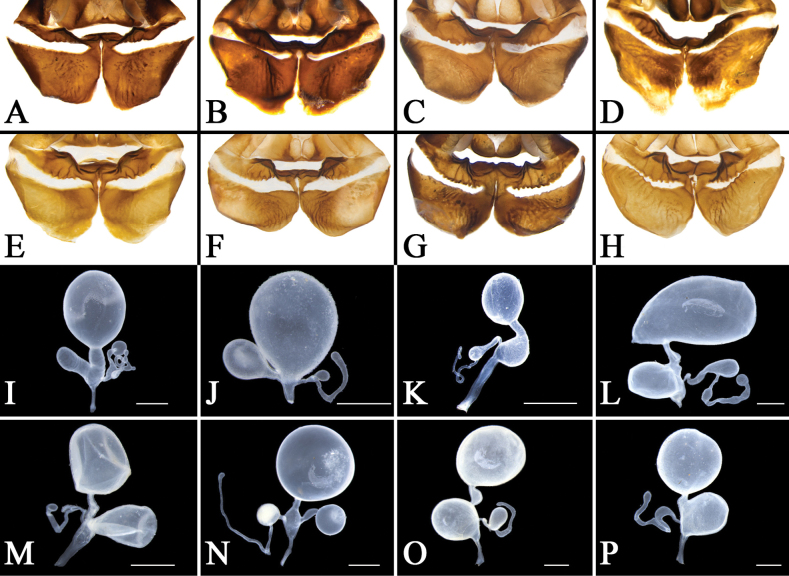
Basivalvula, spermathecal plate and spermatheca of eight *Eupolyphaga* species **A, I***E.bicolor* Han, Che & Wang, sp. nov. **B, J***E.nigra* Han, Che & Wang, sp. nov. **C, K***E.udenostyla* Qiu, 2022 **D, L***E.hupingensis* Qiu, Che & Wang, 2018 **E, M***E.miracidia* Qiu, 2022 **F, N***E.sinensis* (Walker, 1868) **G, O***E.robusta* Qiu, Che & Wang, 2018 **H, P***E.hanae* Qiu, Che & Wang, 2018. Scale bars: 0.05 cm (**I–P**).

Figs 3A–N , 4C, D , 5B, J

##### Type material.

***Holotype***: China • male; Guangxi Zhuang Autonomous Region, Chongzuo City, Longzhou County, Zhubu Village, Buji Reservoir; 7 Jul. 2023; Wei Han, Xin-Ran Li leg. ***Paratypes***: China • 3 males, 1 female & 16 nymphs, same collection data as holotype.

##### Diagnosis.

This species is almost black and is most similar to *E.robusta*. However, the abdomen of this species is unevenly scattered with some fulvous markings, whereas the abdomen of the latter is orange-yellow overall or dark yellow only on the two terminal segments. In addition, the middle part of terga of females of this species is slightly dark yellowish brown, whereas the terga of females of the latter is completely black; although serrations on the keel of both species are strongly curved, there are gaps between the serrated projections of the ootheca of this species, whereas there are almost no gaps in the latter.

##### Description.

**Male holotype. *Measurements* (mm).** Overall length (including tegmen): 27.73; body length: 19.67; body width (tegmina not included): 10.03; tegmen length × width: 23.98 × 9.65; pronotum length × width: 8.13 × 4.79.

***Coloration*.** Head and most of the face black. Ocelli and antennal sockets white. Antennae blackish brown. Ante-clypeus, basal part of the labrum, and a portion of the palate yellow (Fig. 3A, B, G). Pronotum, tegmina, hind wings and legs black. Pulvilli and arolia white. Abdomen black, with some fulvous markings (Fig. 3A, B).

***Body*. *Head***: Sub-rounded, hidden under the pronotum. Interocular space narrower than the distance between the ocelli, and the latter narrower than the distance between the antennal sockets. Ocelli distinct. Ocelli ridge curved, with a row of setae on the upper edge. Clypeus developed (Fig. 3B, G). ***Pronotum***: Transverse oval, widest point near the middle. Anterior whitish margin indistinct. Surface covered with short setae (Fig. 3E). ***Tegmina and hind wings***: Nearly unicolored, with a few plaques on both sides (Fig. 3A). ***Legs***: Slender. Front femur type C_1_. Pulvilli and arolia present (Fig. 3B). ***Abdomen***: Supra-anal plate transverse, pubescent, posterior margin protruded. Paraprocts simple. Cerci pubescent. Posterior margin and lateral margins of subgenital plate densely setose, hind margin flat. Styli small, the right one bigger than the left (Fig. 3B, I–L). ***Genitalia***: The basal part of L1 prolonged, and the two hind lobes robust. L2 curved. Genital hook (L3) long and robust, the hook part curved. Right phallomere smaller than the left phallomere. R2 simple, divided into two chunks. R3 broad and concave (Fig. 3K, L).

**Female paratype.** Body length: 27.79; body width: 19.12; pronotum length × width: 13.51 × 7.15.

***Coloration*.** Terga dark yellowish brown to black. Sterna nearly black. Vertex and face black. Ocelli yellow. Basal part of labrum black. Distal part of labrum and ante-clypeus yellow. Legs black (Fig. 3C, D, F, H).

The widest point of pronotum near the hind margin (Fig. 3F). Ocelli distinct. Interocular space larger than the distance between antennal sockets, and the latter larger than the distance between ocelli (Fig. 3H). Arolia and pulvilli absent. Posterior margin of the supra-anal plate (TX) emarginated medially. Cerci short, not exceeding the posterior margin of supra-anal plate. Paraprocts (pp.) pubescent, curved hook-like extensions long. The two median sclerites irregularly shaped (Fig. 3C). Paratergites (pt.) banded, terminally bifurcated. Crosspiece (cp.) broad, the small protrusion short and wide. Posterior lobes of valvifer II (p.l.) short, terminal rounded. Spermathecal plate (sp.pl.) narrow and concave in the middle, the two lobes expanded in the middle (Fig. 5B). Spermatheca (sp.) consists of two distinct large ampullas, the bifurcated duct slightly expanded in the middle (Fig. 5J). Two lobes of basivalvula (bsv.) nearly triangular, with a flat anterior margin and a curly lateral margin. Vestibular sclerite (vst.s) shaped like the letter “W”, with widened apex on both sides and a robust, short protrusion in the middle. Subgenital plate (SVII) densely setose, posterior margin protruded (Fig. 3D).

**Nymph.** Similar to the female.

**Ootheca.** Yellowish brown. The longitudinal lines distinct. Serrations on the keel large and curved. The space between the serrations of the curved portion distinct. Respiratory canals well developed (Fig. 3M, N).

##### Natural history.

Found in soft, dry soil under the cliffs near the reservoir.

##### Etymology.

The species epithet is from the Latin *niger* indicating its black tegmina.

#### ﻿ Eupolyphaga udenostyla

Taxon classificationAnimaliaBlattodeaCorydiidae

Qiu, 2022

88E09EFF-836B-5DB6-952C-1BC5D7120449

Figs 4E, F , 5C, K

Eupolyphaga udenostyla Qiu in Han et al. 2022: 75.

##### Material examined.

• 1 female; Sichuan Prov., Aba Prefecture, Wenchuan County, Keku Township; 7 Aug. 2019; Wei Han, Huan-Yu Ren leg • 1 female; same collection data as above, but 5 Oct. 2019; Lu Qiu leg • 4 females; Sichuan Prov., Aba Prefecture, Wenchuan County, mountains behind the 5.12 Wenchuan Earthquake Memorial Museum; Jul.–Aug. 2019; Qi Li leg.

##### Description on the female characters.

Supra-anal plate (TX) black, densely covered with long brown setae. Paraprocts (pp.) pubescent, with thin and short curved hook-like extensions. Cerci short, not exceeding the posterior margin of the supra-anal plate. Paratergites (pt.) banded. Crosspiece (cp.) nearly transparent and the protrusion small. The first valvule (v.I) long, basal part connected to the spermathecal plate (sp.pl.). Basal of the second valvule (v.II) broad, terminal sharp. Basal part of the third valve (v.III) enlarged. Posterior lobes of valvifer II (p.l.) slightly sclerotized. The spermathecal plate narrow, arched in the middle. The anterior margin and hind margin of the two lobes have irregular protrusions. The spermatheca (sp.) consists of two distinct large ampullas. The basal ampulla connected to a long spermathecal duct; the middle part of the duct has a small globular enlargement. Basivalvula (bsv.) broad, with a flat anterior margin and a curly lateral margin. Vestibular sclerite (vst.s) shaped like the letter “W”, apically expanded in both sides, the tip of the central protuberance emarginated. The subgenital plate (SVII) densely setose, the terminal part of the posterior margin emarginated.

#### ﻿ Eupolyphaga hupingensis

Taxon classificationAnimaliaBlattodeaCorydiidae

Qiu, Che & Wang, 2018

3F76BFAE-B7DE-59F1-AC18-AC1ACADB0333

Figs 4G, H , 5D, L

Eupolyphaga hupingensis Qiu, Che & Wang, 2018: 18; Qiu et al. 2019: 11 (catalogue); Han et al. 2022: 88.

##### Material examined.

• 1 female; Hunan Prov., Shaoyang City, Xinning County, Huanglong Town, Lizhu Village, Shunhuang Mountain, Zihua Ping; 24–25 May 2022; Lu Qiu leg.

##### Description of the female characters.

Supra-anal plate (TX) black and densely covered with setae, the posterior margin slightly flat. Paraprocts (pp.) pubescent, curved hook-like extensions thin and long. The two median sclerites irregularly shaped. Cerci short, not exceeding the posterior margin of supra-anal plate. Paratergites (pt.) long and banded. Crosspiece (cp.) weakly sclerotized, barely visible. Posterior lobes of valvifer II (p.l.) short and robust. Spermathecal plate (sp.pl.) concave in the middle, with two narrow lobes. Spermatheca (sp.) consists of two distinct and large ampullas, the terminal ampulla larger. The duct bifurcated near the basal ampulla, and the bifurcated duct expands into a small ball in the middle. Basivalvula (bsv.) transverse, the two lobes nearly triangular, and the lateral margin curled. Subgenital plate (SVII) setose, the terminal part of the posterior margin flat and emarginated medially.

#### ﻿ Eupolyphaga miracidia

Taxon classificationAnimaliaBlattodeaCorydiidae

Qiu, 2022

0AC11280-6B17-593F-8CCB-B4CF5DB32E99

Figs 4I, J , 5E, M

Eupolyphaga miracidia Qiu in Han et al. 2022: 73.

##### Material examined.

• 5 females; Hubei Prov., Xiangyang City, Maqiao Township, roadside of Ganxigou, 480–600 m; 13 Jul. 2017; Lu Qiu leg.

##### Description of the female characters.

Supra-anal plate (TX) dark yellowish brown and densely covered with setae, posterior margin slightly protruded. Paraprocts (pp.) pubescent, curved hook-like extensions short. The two median sclerites irregularly shaped. Cerci short, not exceeding the posterior margin of supra-anal plate. Paratergites (pt.) long and banded. Crosspiece (cp.) nearly transparent, the protrusion long. Posterior lobes of valvifer II (p.l.) slightly sclerotized, two lobes long and curved. Spermathecal plate (sp.pl.) narrow, concave in the middle. The two lobes expanded, with irregular protrusions. Spermatheca (sp.) consists of two distinct, large ampullas. The basal ampulla connected to a long spermathecal duct, which is bifurcated in the middle. The terminal part of the duct slightly expanded. Basivalvula (bsv.) broad, with a relatively flat anterior margin and a curly lateral margin. Vestibular sclerite (vst.s) shaped like the letter “W”, with expanded and elongated ends on both sides. Subgenital plate densely setose, the terminal part of the posterior margin protruded and emarginated medially.

#### ﻿ Eupolyphaga sinensis

Taxon classificationAnimaliaBlattodeaCorydiidae

(Walker, 1868)

28CCBD8B-9FC7-576B-BAA6-1A37AA4589C3

Figs 4K, L , 5F, N

Polyphaga sinensis Walker, 1868: 14.
Homoeogamia sinensis : Saussure 1869: 282; Hollier et al. 2020: 347. Synonymized by Qiu et al. 2018.
Heterogamia sinensis : Dohrn 1888: 132.
Heterogamia dohrniana Saussure, 1893: 309; Hollier et al. 2020: 345.
Polyphaga limbata Kirby, 1903: 379.
Eupolyphaga sinensis : Chopard 1929: 262; Qiu et al. 2018: 5 (revision); Qiu et al. 2019: 11 (checklist); Han et al. 2022: 84.

##### Material examined.

• 2 females; Beijing City, Haidian District, Beijing Xishan National Forest Park; 28 Apr. 2015; Bing–Qiang Wang leg • 1 female; Anhui Prov. Hefei City, Binhu County; 3 Oct. 2018; Lin Zhou leg • 1 female; Jiangsu Prov., Nanjing City, Xuanwu District, Zijin Mountain, Zhongshan Mausoleum; 18 Jul. 2021; Ya-Ning Sun, Yi-Fan Zhao leg.

##### Description on the female characters.

Supra-anal plate (TX) dark yellowish brown and densely covered with setae, the posterior margin protruded medially. Paraprocts (pp.) pubescent, curved hook-like extensions thin and long. The two median sclerites irregularly-shaped. Cerci short, not exceeding the posterior margin of supra-anal plate. Paratergites (pt.) long and banded. Crosspiece (cp.) nearly transparent, the protrusion robust. Posterior lobes of valvifer II (p.l.) weakly sclerotized. Spermathecal plate (sp.pl.) broad, distinctly concave in the middle, two lobes foliated. Spermatheca (sp.) consists of four distinct, large ampullas. The terminal ampulla abnormally enlarged, with a bifurcated catheter attached to one side of the ampulla. Basivalvula (bsv.) transverse, with two long and narrow lobes, the lateral margin curly. Vestibular sclerite (vst.s) shaped like the letter “W”. The three protrusions almost identical in height. Terminal of both sides’ protrusion expanded. Subgenital plate (SVII) densely setose, posterior margin protruded and the terminal part emarginated medially.

#### ﻿ Eupolyphaga robusta

Taxon classificationAnimaliaBlattodeaCorydiidae

Qiu, Che & Wang, 2018

19384B92-7CAC-5065-A62E-8312682D922A

Figs 4M, N , 5G, O

Eupolyphaga robusta Qiu, Che & Wang, 2018: 19; Qiu et al. 2019: 11 (catalogue); Han et al. 2022: 86.

##### Material examined.

• 1 female; Sichuan Prov., Aba Prefecture, Wenchuan County, Miansi Town; 29 March 2020; Jian-Yue Qiu leg • 1 female; Sichuan Prov., Aba Prefecture, Maoxian County, Nanxin Town, Miancu Village; 7 Aug. 2019; Zong-Qing Wang, Lu Qiu, Wei Han, Huan-Yu Ren leg • 1 female; Sichuan Prov., Aba Prefecture, Maoxian County, Xiaomiao Mountain; 6 Aug. 2019; Lu Qiu, Wei Han, Huan-Yu Ren leg.

##### Description of the female characters.

Supra-anal plate (TX) black and covered with setae, posterior margin slightly protruded in the middle. Paraprocts (pp.) pubescent, the curved hook-like extensions long. The two median sclerites irregularly-shaped. Cerci short, not exceeding the posterior margin of supra-anal plate. Paratergites (pt.) long and banded. Crosspiece (cp.) well-sclerotized and the protrusion robust. Posterior lobes of valvifer II (p.l.) short. Spermathecal plate (sp.pl.) narrow and slightly concave in the middle, with two lobes that have distinct arch in the middle. Spermatheca (sp.) consists of two distinct, large ampullas, the terminal ampulla bigger, and the duct connecting the two ampullas slightly expanded. The ampulla near the base also connected to a duct that expands into a small ball in the middle. Basivalvula (bsv.) transverse, two lobes wide, lateral margins curly. Vestibular sclerite (vst.s) shaped like the letter “W”, slightly expanded at the terminal of both sides’ protrusions. The middle protrusion forked at the tip. Subgenital plate (SVII) densely setose, the posterior margin protruded and emarginated terminally.

#### ﻿ Eupolyphaga hanae

Taxon classificationAnimaliaBlattodeaCorydiidae

Qiu, Che & Wang, 2018

78CFD24F-7CFF-5728-B861-C75199C1E6CF

Figs 4O, P , 5H, P

Eupolyphaga hanae Qiu, Che & Wang, 2018: 42; Qiu et al. 2019: 11 (checklist); Han et al. 2022: 84.

##### Material examined.

• 5 females; Chongqing City, Beibei District, Jinyun Mountain, Southwest Bureau Statue; 10 Jul. 2021; Wei Han leg • 3 females; Sichuan Prov., Suining City, Shehong County, Fuxing Town, Taixing Township, Laogangmo Village; 8 Mar. 2016; Lei Wang leg • 1 female; Sichuan Prov., Jiangjin District, Simian Mountain, Shunzigou; 6 Mar. 2016; Jian-Yue Qiu, Hao Xu leg • 1 female; Sichuan Prov., Mianyang City, Jiangyou County, Qianyuan Mountain, Jinguangdong; 16 Jan. 2022; Hao Xu, Xin-Yuan Zhang leg.

##### Description of the female characters.

Supra-anal plate (TX) reddish brown and densely covered with setae. The posterior margin flat. Paraprocts (pp.) pubescent, curved hook-like extensions short. The two median sclerites irregularly shaped. Cerci short, not exceeding the posterior margin of supra-anal plate. Paratergites (pt.) long and banded. Crosspiece (cp.) nearly transparent, the protrusion long and robust. Posterior lobes of valvifer II (p.l.) slightly sclerotized, two lobes long and curved, with poorly-defined edges. Spermathecal plate (sp.pl.) broad and concave in the middle, two lobes with distinct cone-shaped protrusions. The posterior margin of the lobe with irregular protrusions. Spermatheca (sp.) consists of two distinct, large ampullas. The basal ampulla connected to a long spermathecal duct, the duct slightly expanded in the middle and terminal portions. Basivalvula (bsv.) transverse, anterior margin elongated terminally, the lateral margin curled. Vestibular sclerite (vst.s) shaped like the letter “W”, expanded at the terminal of both sides. Subgenital plate (SVII) densely setose, posterior margin protruded and the terminal part emarginate medially.

#### ﻿Genus Pseudoeupolyphaga

Taxon classificationAnimaliaBlattodeaCorydiidae

Qiu & Che, 2024

5E22E002-C08B-59D7-81F7-B50578FC703B

Pseudoeupolyphaga Qiu & Che in Han et al. 2024: 165.

##### Type species.

*Polyphagayunnanensis* Chopard, 1922, by original designation.

##### Supplementary diagnosis.

**Figure 6.**
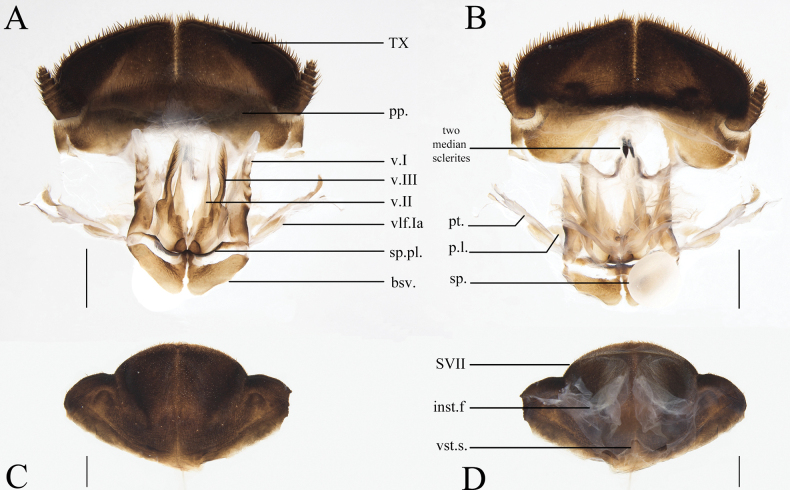
The supra-anal plate, subgenital plate, female external genitalia and spermatheca of the genus *Pseudoeupolyphaga*, using *P.yunnanensis* (Chopard, 1922) as an exemplar **A** supra-anal plate and female external genitalia, ventral view **B** supra-anal plate and female external genitalia, dorsal view **C** subgenital plate, ventral view **D** subgenital plate, dorsal view. Scale bars: 0.1 cm (**A–D**). Abbreviations: bsv. basivalvula, p.l. posterior lobes of valvifer II, pp. paraprocts, pt. paratergites, sp. spermatheca, sp.pl. spermathecal plate, SVII subgenital plate, TX supra-anal plate, v.I first valvule, v.II second valve, v.III third valve, vlf.Ia first valvifer arm, vst.s vestibular sclerite.

Following anatomical examination of specimens representing 15 species and subspecies, no noteworthy variations were discerned in the sclerites of female external genitalia and the shape of spermathecae across different species within this genus. Consequently, detailed descriptions of female external genitalia and spermathecae for these species were omitted, and instead, a summary diagnosis encompassing the genus is provided (Fig. 6). Comprehensive information and illustrations of the anatomical samples are available in the supplementary material (Suppl. materials 1, 2). Paratergites (pt.) banded or lamellar. Crosspiece (cp.) indistinct or distinct, with a small protuberance that points towards the posterior lobes of valvifer II (p.l.). The posterior lobes of valvifer II fuse with the anterior arch (aa.) forming a circinate structure. Posterior lobes of valvifer II well-sclerotized or not, curved apically. The first valvule (v.I) long, slightly curved, with more pronounced lateral sclerotization. Basal part of valvifer II (v.II) and valvifer III (v.III) enlarged. The spermathecal plate (sp.pl.) well-sclerotized, narrow, depressed downward in the middle. Basivalvula (bsv.) symmetrical, with two lobes narrow. Each lobe with curved anterior margins, curly lateral margins, and round posterior margins. The spermatheca (sp.) consists of a large spherical ampulla and a short spermathecal duct. With or without a curved and elongated duct attached to the ampulla. The vestibular sclerite (vst.s) shaped like the letter “W”, with three protrusions. The subgenital plate (SVII) densely setose, posterior margin bulging and protruding, with middle part slightly concave inward or not.

#### ﻿ Pseudoeupolyphaga flava

Taxon classificationAnimaliaBlattodeaCorydiidae

Han, Che & Wang sp. nov.

32920712-FAD3-5500-BF7D-B83CED1D6F96

https://zoobank.org/93E7B897-8469-4081-84B6-8D290B586FDD

Fig. 7A–L

##### Type material.

***Holotype***: China • male; Yunnan Province, Lijiang City, Yongsheng County, Liude Village, G353 roadside in dry soil; 9 Jul. 2021; Lu Qiu, Hao Xu leg. ***Paratypes***: China • 2 males, 1 female & 7 nymphs, same collection data as holotype.

##### Diagnosis.

This species can be easily distinguished from others by its bright yellowish abdomen, present in both males and females. In addition, the males of this species have large patches in the middle of their tegmina, which is distinctly different from other congeneric species.

##### Description.

**Holotype. *Measurements* (mm).** Overall length (including tegmen): 25.58; body length: 18.24; body width (tegmina not included): 9.21; tegmen length × width: 21.41 × 7.40; pronotum length × width: 6.95 × 3.76.

**Figure 7.**
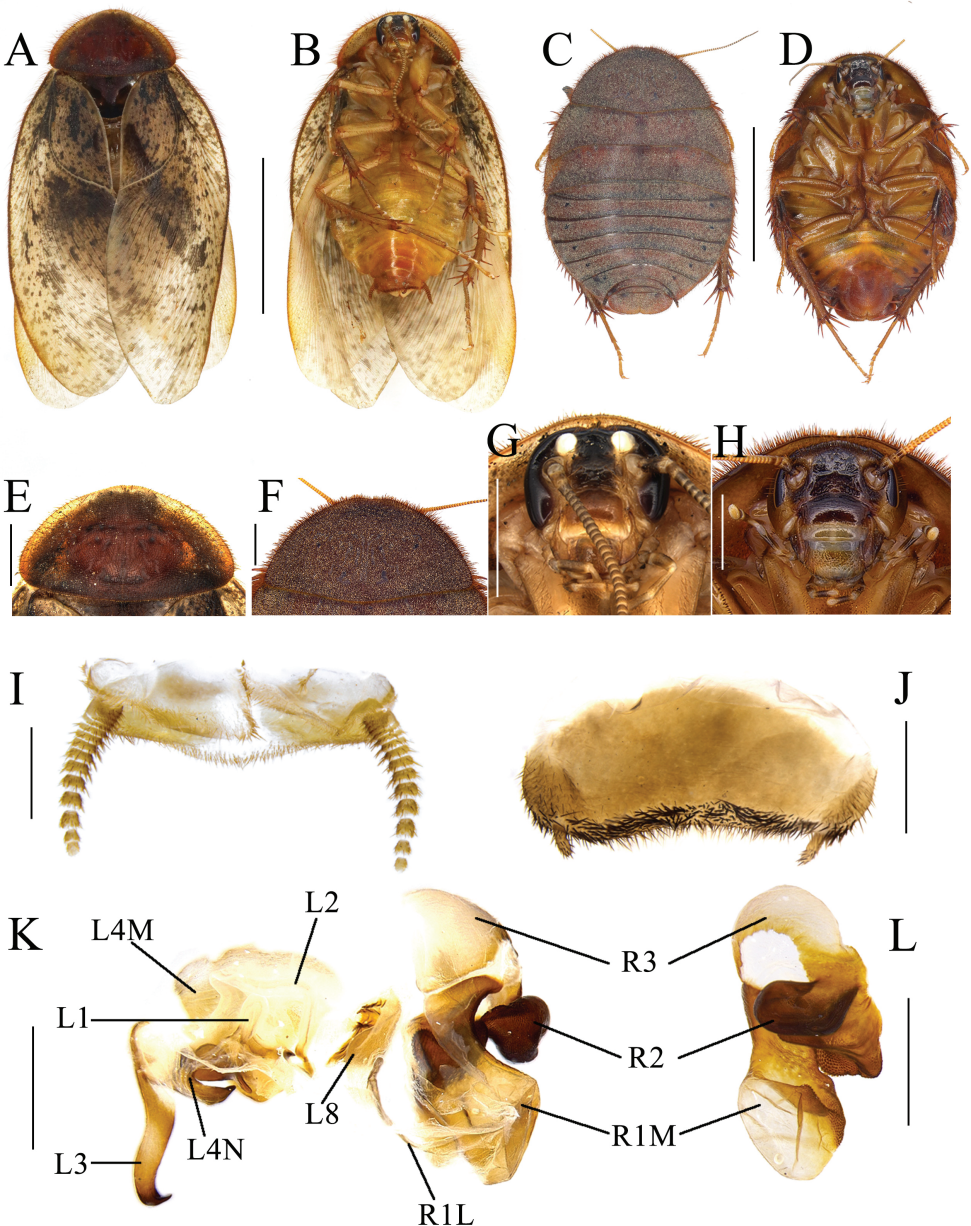
*Pseudoeupolyphagaflava* Han, Che & Wang, sp. nov. **A, B, E, G, I–L** male holotype **C, D, F, H** female paratype **A** habitus, dorsal view **B** habitus, ventral view **C** habitus, dorsal view **D** habitus, ventral view **E** pronotum, dorsal view **F** pronotum, dorsal view **G** head, ventral view **H** head, ventral view **I** supra-anal plate, ventral view **J** subgenital plate, ventral view **K** genitalia, dorsal view **L** right phallomere, right-ventral view. Scale bars: 1.0 cm (**A–D**); 0.2 cm (**E–H**); 0.1 cm (**I–L**).

***Coloration*.** Body mostly yellow (Fig. 7A, B). Pronotum dark yellowish brown to reddish brown, anterior margin white, with short yellow setae (Fig. 7E). Tegmina pale gray, with densely darkish brown maculae. Hind wings nearly transparent, also with densely pale-colored maculae (Fig. 7A, B). Head black. Ocelli white. Antennae brownish yellow. Forehead black. Ante-clypeus white, post-clypeus yellowish brown. Labrum pale yellowish brown (Fig. 7G). Legs yellow, tibia, tarsi, and ante-tarsi yellowish brown. Pulvilli and arolia white. Abdomen yellow, distal part slightly darker in color (Fig. 7B).

***Body*. *Head***: Sub-rounded, hidden under pronotum. Eyes developed, ocelli bulging round and protruded. Interocular space narrower than the distance between ocelli, the latter narrower than the distance between antennal sockets. Ocelli ridge indistinct, with a row of setae on the upper edge. Clypeus developed (Fig. 7G). ***Pronotum***: Transverse oval, widest near the hind margin. Surface with short setae. Anterior whitish margin narrow, clearly demarcated from the yellowish-brown area, with symmetrical dark protrusions in the center (Fig. 7E). ***Tegmina and hind wings***: Maculae dense and of different size. A large fused brown macula located in the center (Fig. 7A). ***Legs***: Slender, front femur type C_1_. Pulvilli and arolia present (Fig. 7B). ***Abdomen***: Supra-anal plate transverse, pubescent, posterior margin slightly protruded medially. Paraprocts simple. Cerci long. Subgenital plate with short setae, hind margin slightly concave medially. Left stylus shorter than the right one (Fig. 7I, J). ***Genitalia***: Right phallomere bigger than the left phallomere. L1 basally prolonged, two hind lobes weakly sclerotized. L2 arching, curved. Genital hook (L3) short and robust, the hook small. L4M broadly lamellate; pda subtriangular, paa broad. L5 subelliptic. L8 basally dilated, tip with a protrusion. Right phallomere long. R1M stout. R1L banded, elongate. R2 divided into two chunks, the basal one more rounded, the upper one with a flatter anterior margin and a protruded prolonged right posterior lateral angle. R3 thin, convex, and irregular (Fig. 7K, L).

**Male paratypes.** Similar to the holotype.

**Female paratype.** Body length: 20.20 mm; body width: 13.00 mm; pronotum length × width: 10.61 × 6.53 mm.

***Coloration*.** Terga yellowish brown to reddish brown, margins with yellowish brown setae (Fig. 7C). Sterna yellow, the distal part slightly darker (Fig. 7D). Head black. Ocelli white. Ante-clypeus sub-transparent, pale gray. Post-clypeus blackish brown. Basal part of labrum pale gray (Fig. 7H). Legs yellow, tibia nearly black. Spines dark yellowish brown to black (Fig. 7C, D).

***Body*.** The widest point of pronotum near the hind margin, anterior whitish margin indistinct (Fig. 7F). Ocelli indistinct, degraded to two small white spots. Interocular space bigger than the distance between ocelli, and almost equal to the distance between antennal sockets (Fig. 7H). Front femur type C_1_. Arolia and pulvilli absent. Supra-anal plate densely covered with long yellowish brown setae, posterior margin slightly convex, slightly emarginated medially. Cerci short and robust, not exceeding posterior margin of supra-anal plate. Posterior margin of subgenital plate protruded, emarginated medially (Suppl. material 1: fig. S1A).

**Nymph.** Similar to the female.

**Ootheca.** Unknown.

##### Etymology.

The species epithet is derived from the Latin word *flavus*, referring to the yellowish abdomen of both males and females.

##### Remark.

The interspecific genetic distance between this species and the other species within this genus ranges from 10.62% to 20.39%, providing support for the classification of this species as a novel taxon.

#### ﻿ Pseudoeupolyphaga deficiens

Taxon classificationAnimaliaBlattodeaCorydiidae

Han, Che & Wang sp. nov.

F8CF5A67-CDE9-5B1E-84F9-119A25C1F0C9

https://zoobank.org/BC9BB5BD-69BB-4C77-82CC-2B3074E26956

Figs 8A–L , 15A, J

##### Type material.

***Holotype***: China • male; Sichuan Province, Aba Prefecture, Heishui County, entrance to Dagu Glacier; 22 Jun. 2021; Lu Qiu, Hao Xu leg. ***Paratypes***: China • 1 female & 1 ootheca, same collection data as holotype • 1 female & 20 nymphs, Sichuan Province, Mao County, Cuoji Mountain; 6 Aug. 2019; Lu Qiu leg.

##### Diagnosis.

This species is distinguishable from others by the broad anterior white margin of the pronotum and the absence of a distinct boundary between the markings on the tegmina in males. In addition, the surface of the ootheca of this species is unusually smooth, with serrated protuberances and blunt tips.

##### Description.

**Holotype. *Measurements* (mm).** Overall length (including tegmen): 30.86; body length: 18.62; body width (tegmina not included): 10.34; tegmen length × width: 26.28 × 10.49; pronotum length × width: 7.51 × 3.91.

**Figure 8.**
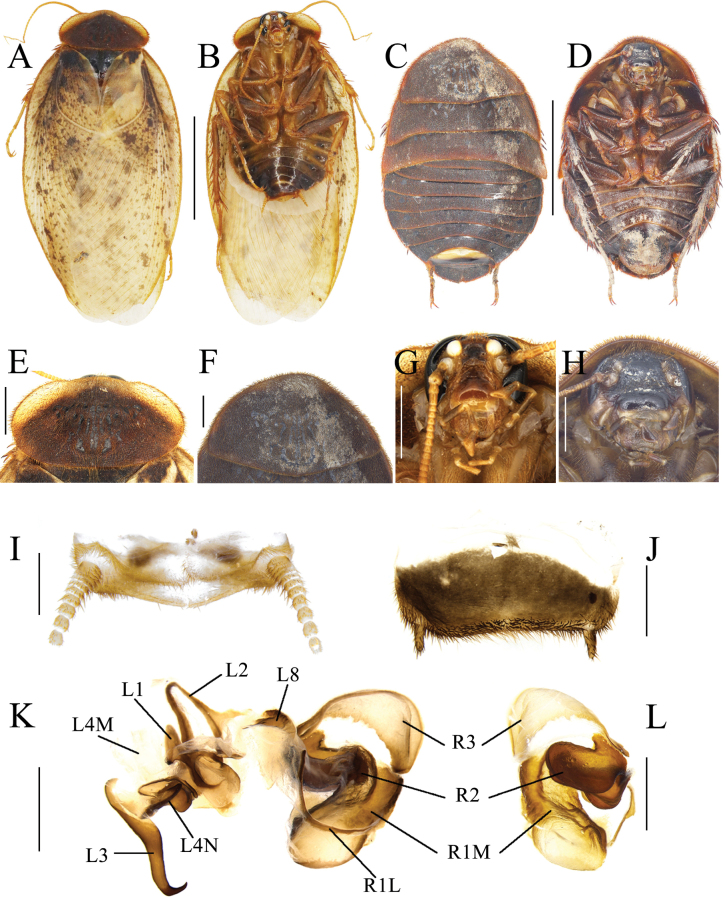
*Pseudoeupolyphagadeficiens* Han, Che & Wang, sp. nov. **A, B, E, G, I–L** male holotype **C, D, F, H** female paratype. **A** habitus, dorsal view **B** habitus, ventral view **C** habitus, dorsal view **D** habitus, ventral view **E** pronotum, dorsal view **F** pronotum, dorsal view **G** head, ventral view **H** head, ventral view **I** supra-anal plate, ventral view **J** subgenital plate, ventral view **K** genitalia, dorsal view **L** right phallomere, right-ventral view. Scale bars: 1.0 cm (**A–D**); 0.2 cm (**E–H**); 0.1 cm (**I–L**).

***Coloration*.** Body yellowish brown (Fig. 8A, B). Pronotum reddish brown, covered with yellowish setae, anterior margin white (Fig. 8E). Tegmina pale yellow, with brown maculae. Wings nearly transparent (Fig. 8A, B). Face yellow. Antennae yellow. Eyes black. Ocelli white. Middle of forehead with a dark brown macula. Ante-clypeus pale yellow, post-clypeus yellowish brown. Labrum yellow (Fig. 8G). Legs yellowish brown, tibia dark yellowish brown. Pulvilli and arolia white. Abdomen yellowish brown and gradually darkening toward the distal abdomen (Fig. 8B).

***Body*. *Head***: Sub-rounded, hidden under pronotum. Eyes and ocelli developed. Ocelli ridge slightly curved, with a row of setae on the upper edge. Interocular space narrower than the distance between ocelli, the latter narrower than the distance between antennal sockets. Clypeus developed (Fig. 8G). ***Pronotum***: Transverse oval, widest near the middle. Surface densely covered with short setae, center part with symmetrical black stripe. Anterior whitish margin broad, clearly delineated from reddish brown areas (Fig. 8E). ***Tegmina and hind wings***: Markings varied in size and denser near the base of the tegmina (Fig. 8A, B). ***Legs***: Slender, front femur type C_1_. Pulvilli and arolia present (Fig. 8B). ***Abdomen***: Supra-anal plate transverse, pubescent, middle part of posterior margin slightly protruded. Paraprocts simple. Subgenital plate with short setae, hind margin flat. Styli long (Fig. 8I, J). ***Genitalia***: Well-sclerotized. Right phallomere bigger than the left phallomere. Anterior protrusion of L1 long and sharp. L2 arching curved. Genital hook (L3) robust, curved hook section nearly right-angled. L4M broad lamellate. The protrusion of pda and paa broad. L7 sub-membranous, ovoid. L8 irregular, subtriangular. R1M stoutly expanded terminally, R1L elongate and banded. R2 divided into two chunks of approximate size, narrowly spaced, with rounded margins. R3 broadly concave (Fig. 8K, L).

**Female paratype (same locality as holotype).** Body length: 20.96 mm; body width: 13.82 mm; pronotum length × width: 10.75 × 6.46 mm.

***Coloration*.** Terga dark yellowish brown (Fig. 8C). Head black. Antennae yellow. Ocelli white. Ante-clypeus yellowish white. Post-clypeus black. Labrum yellowish brown (Fig. 8H). Legs dark yellowish brown, with large dark brown patches. Spines on the leg reddish brown, terminal nearly black. Sterna dark yellowish brown, margins and both sides nearly blackish brown; middle part slightly lighter, yellowish brown (Fig. 8D).

***Body***. The widest point of pronotum near the hind margin, middle part with symmetrical black dark stripe, anterior whitish margin indistinct (Fig. 8F). Ocelli indistinct, degraded to two white spots. Interocular space almost equal to the distance between antennal sockets, both bigger than the distance between ocelli (Fig. 8H). Front femur type C_1_. Arolia and pulvilli absent. Supra-anal plate densely covered with yellowish brown setae, posterior margin convex, middle part slightly emarginated. Cerci short and robust, not exceeding posterior margin of supra-anal plate. Posterior margin of subgenital plate protruded, emarginated medially (Suppl. material 1: fig. S1B).

**Nymph.** Similar to the female.

**Ootheca.** Reddish brown. Surface with densely parallel longitudinal lines. Ridges of serrated protuberances densely arranged with blunt tips. No respiratory canals (Fig. 15A, J).

##### Etymology.

The species epithet is derived from the Latin word *deficiens*, to refer to the markings on the tegmina that lack distinct boundaries.

##### Remark.

The genetic distance from other species was 8.39%–20.30%, which also provides evidence supporting the description of this new species.

#### ﻿ Pseudoeupolyphaga magna

Taxon classificationAnimaliaBlattodeaCorydiidae

Han, Che & Wang sp. nov.

BC66DBFD-00DA-5AD8-84DC-122794A78FBA

https://zoobank.org/72A2A7E7-49EB-442A-B2C5-121C8180C1D6

Fig. 9A–L

##### Type material.

***Holotype***: China • male; Sichuan Province, Aba Prefecture, Jinchuan County, Guanyinqiao Township; 2020; Jian-Yue Qiu leg. ***Paratype***: China • 1 female, same collection data as holotype.

##### Diagnosis.

The males of this species closely resemble *P.yunnanensis*, but are significantly larger than all other species in this genus as currently known, and can be distinguished accordingly.

##### Description.

**Holotype. *Measurements* (mm).** Overall length (including tegmen): 42.44; body length: 27.56; body width (tegmina not included): 14.52; tegmen length × width: 37.40 × 12.60; pronotum length × width: 11.67 × 6.91.

**Figure 9.**
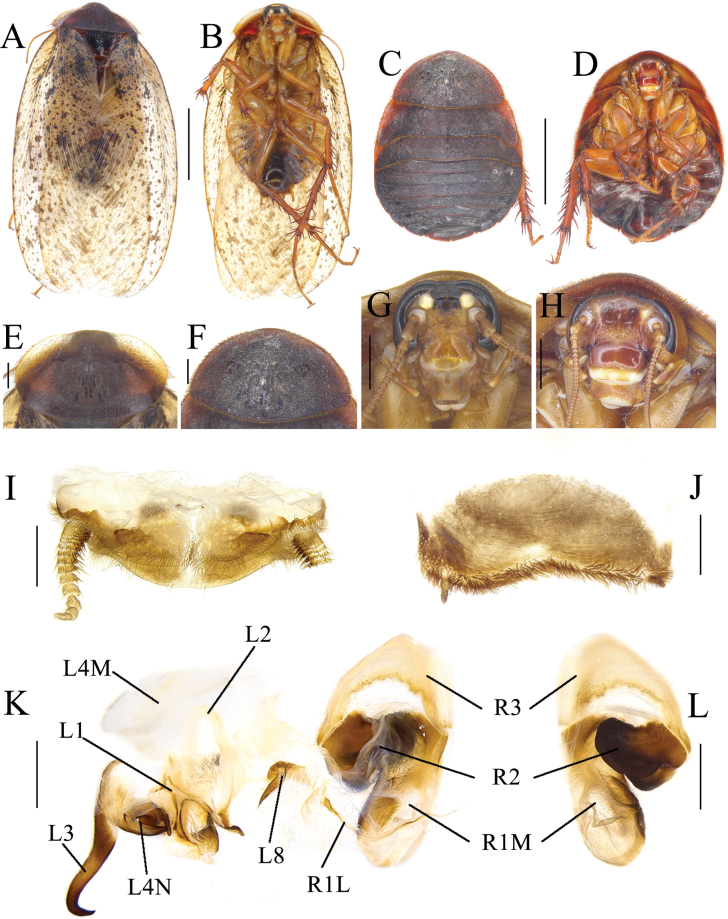
*Pseudoeupolyphagamagna* Han, Che & Wang, sp. nov. **A, B, E, G, I–L** male holotype **C, D, F, H** female paratype **A** habitus, dorsal view **B** habitus, ventral view **C** habitus, dorsal view **D** habitus, ventral view **E** pronotum, dorsal view **F** pronotum, dorsal view **G** head, ventral view **H** head, ventral view **I** supra-anal plate, ventral view **J** subgenital plate, ventral view **K** genitalia, dorsal view **L** right phallomere, right-ventral view. Scale bars: 1.0 cm (**A–D**); 0.2 cm (**E–H**); 0.1 cm (**I–L**).

***Coloration*.** Pronotum yellowish brown, covered with yellowish setae, anterior margin white (Fig. 9A, E). Tegmina subtransparent, densely covered with blackish brown maculae (Fig. 9A). Eyes, vertex, and space between ocelli black. Face yellowish brown. Ocelli, antennal sockets, and ante-clypeus white. Antennae, post-clypeus, labrum, labial palpi and maxillary palpi yellow (Fig. 9G). Legs yellowish brown, tibia and spines dark yellowish brown to black. Pulvilli and arolia white. Sterna yellowish brown, middle and distal part nearly black (Fig. 9B).

***Body*. *Head***: Sub-rounded, hidden under pronotum. Eyes and ocelli developed. Ocelli ridge indistinct, with a row of setae on the upper edge. Interocular space narrower than the distance between ocelli, the latter narrower than the distance between antennal sockets. Clypeus developed (Fig. 9G). ***Pronotum***: Transverse oval, widest near the middle. Sparsely covered with short setae, middle part with symmetrical black stripes. Anterior whitish margin broad on both sides and absent in middle, unclearly delineated from the yellowish-brown areas (Fig. 9E). ***Tegmina and hind wings***: Maculae uniformly distributed and of moderate size. Hind wings nearly transparent, with a few pale brown patches (Fig. 9A). ***Legs***: Slender, front femur type C_1_. Pulvilli and arolia present (Fig. 9B). ***Abdomen***: Supra-anal plate transverse, pubescent, posterior margin slightly protruded medially. Paraprocts simple. Subgenital plate with short setae, hind margin concave in the middle. Styli long (Fig. 9I, J). ***Genitalia***: L1 weakly sclerotized, two posterior lobes diverging widely. L2 arching curved, broad. Genital hook (L3) robust. L4M broad lamellate. Pda and paa developed, protrusions long. L8 irregular. R1M expanded terminally, R1L elongate and banded. R2 with two chunks. R3 broadly concave, sub-transparent (Fig. 9K, L).

**Female paratype.** Body length: 22.31 mm; body width: 17.27 mm; pronotum length × width: 12.88 × 6.82 mm.

***Coloration*.** Terga reddish brown (Fig. 9C). Space between ocelli reddish brown. Antennae yellow. Ocelli, antennal sockets, ante-clypeus as well as upper and lower margins of labrum white. Middle part of labrum yellow. Post-clypeus pale reddish brown (Fig. 9H). Legs yellowish brown, tibia dark yellowish brown. Spines on foot reddish brown to black. Sterna dark reddish brown to black, darker in the middle and edges (Fig. 9D).

The widest point of pronotum near the hind margin, middle part with symmetrical black stripe, anterior whitish margin indistinct (Fig. 9F). Ocelli degraded to two white spots. Interocular space almost equal to the distance between antennal sockets, both larger than the distance between ocelli (Fig. 9H). Front femur type C_1_. Arolia and pulvilli absent. Posterior margin of supra-anal plate protruded, slightly emarginated medially. Cerci short and robust, not exceeding posterior margin of supra-anal plate. Posterior margin of subgenital plate protruded and emarginated medially (Fig. 9C, D).

**Nymph.** Unknown.

**Ootheca.** Unknown.

##### Etymology.

The species epithet is derived from the Latin word *magnus*, referring to the significantly larger male body size than is usual in the genus.

##### Remarks.

The external morphology of this species closely resembles that of *P.yunnanensis*, particularly in the markings on the tegmina and the coloration of abdomen. However, the male of this species is significantly larger than males of the latter. The genetic distance between this species and others ranges from 13.09 to 21.97%, further supporting its status as a new species.

#### ﻿ Pseudoeupolyphaga longiseta

Taxon classificationAnimaliaBlattodeaCorydiidae

Han, Che & Wang sp. nov.

E1FB9C59-2E48-5626-971F-2F7E77765F7E

https://zoobank.org/DF1330DC-C49E-4101-8984-3D8F0C3424BC

Figs 10A–L , 15B, K

##### Type material.

***Holotype***: China • male; Yunnan Province, Diqing Tibetan Autonomous Prefecture, Deqin County, Baimaxueshan Nature Reserve; 27 Jul. 2020; Wei Han, Xin-Xing Luo, Lin Guo leg. ***Paratypes***: China • 1 female & 5 nymphs, same collection data as holotype.

##### Diagnosis.

The male of *P.longiseta* sp. nov. shares similarities with those of *P.simila* and *P.pilosa*, yet the markings on the tegmina of this new species are more densely patterned and darker than in the latter two, particularly near the base of the tegmina. Additionally, some maculae on the tegmina of the new species merged. Unlike males of *P.similar* and *P.pilosa*, which exhibit a yellowish longitudinal line and an interrupted longitudinal line on their abdomen, respectively, this new species lacks a longitudinal line on its abdomen. Additionally, black markings present on the female abdomen of *P.similar* are absent in the females of this new species. In addition, the ootheca of this species has weak serrated protuberances and bluntly rounded tips, which are distinctly different from *P.pilosa*.

##### Description.

**Holotype. *Measurements* (mm).** Overall length (including tegmen): 29.87; body length: 19.45; body width (tegmina not included): 10.38; tegmen length × width: 26.22 × 9.50; pronotum length × width: 7.23 × 4.10.

**Figure 10.**
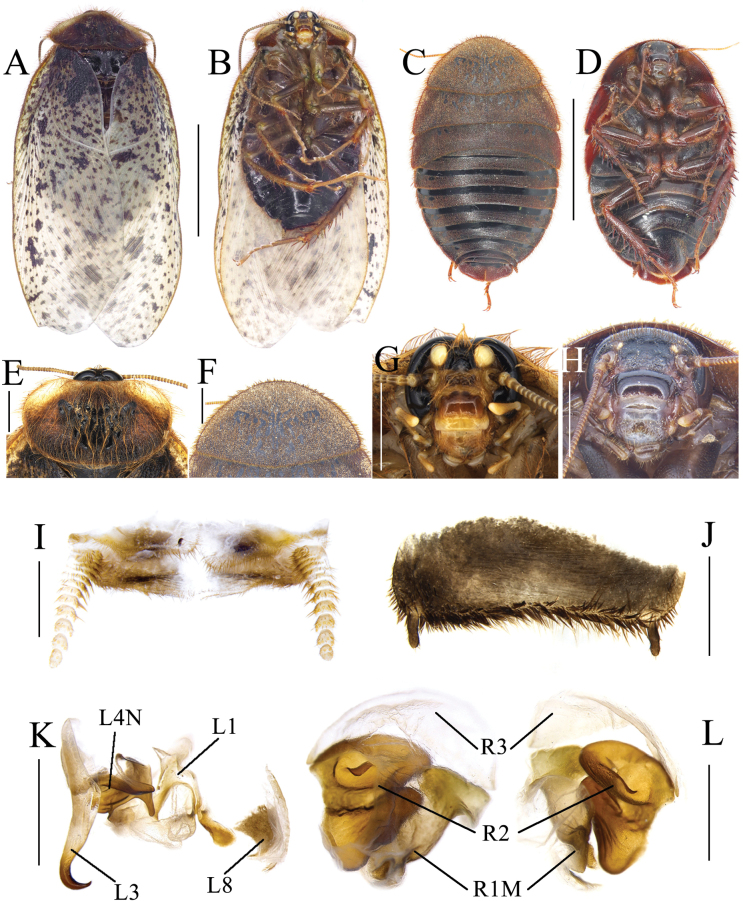
*Pseudoeupolyphagalongiseta* Han, Che & Wang, sp. nov. **A, B, E, G, I–L** male holotype **C, D, F, H** female paratype. **A** habitus, dorsal view **B** habitus, ventral view **C** habitus, dorsal view **D** habitus, ventral view **E** pronotum, dorsal view **F** pronotum, dorsal view **G** head, ventral view **H** head, ventral view **I** supra-anal plate, ventral view **J** subgenital plate, ventral view **K** genitalia, dorsal view **L** right phallomere, right-ventral view. Scale bars: 1.0 cm (**A–D**); 0.2 cm (**E–H**); 0.1 cm (**I–L**).

***Coloration*.** Pronotum yellowish brown, covered with long yellowish setae, anterior margin white (Fig. 10A, E). Tegmina pale gray, densely covered with black maculae. Hind wings subtransparent, with pale blackish brown maculae (Fig. 10A). Eyes and space between ocelli black. Ocelli yellowish white. Antennae and labrum yellowish brown. Space between antennal sockets yellowish brown, middle with a black marking. Ante-clypeus, labial palpi and maxillary palpi yellowish white. Post-clypeus dark yellowish brown (Fig. 10B, G). Legs yellowish brown, outside of tibia nearly blackish brown. Pulvilli and arolia white. Sterna nearly black (Fig. 10B).

***Body*. *Head***: Sub-rounded, not completely hidden under pronotum. Eyes and ocelli developed. Ocelli ridge narrow, with a row of setae on the upper edge. Interocular space narrower than the distance between ocelli, the latter narrower than the distance between antennal sockets. Clypeus developed (Fig. 10G). ***Pronotum***: Transverse oval, widest near the anterior margin. Densely covered with long setae, central part with a symmetrical black stripe. Anterior whitish margin broad, clearly delineated from the yellowish-brown areas (Fig. 10E). ***Tegmina and hind wings***: Tegmina densely covered with maculae. The markings near the base of the tegmina unusually dense and continuous. Hind wing nearly transparent, with a few pale brown patches (Fig. 10B). ***Legs***: Slender, front femur type C_1_, pulvilli and arolia present (Fig. 10B). ***Abdomen***: Supra-anal plate transverse, narrow and pubescent, posterior margin slightly protruded. Paraprocts simple. Margins of subgenital plate densely covered with setae, hind margin slightly concave in the middle. Styli long (Fig. 10I, J). ***Genitalia***: L1 weakly sclerotized, the left protuberance sharp, two posterior lobes diverge widely. Genital hook (L3) robust. L4M broad lamellate. Pda and paa developed, protrusions long. L8 irregular, flaky. R1M with slightly flattened posterior margin. R1L elongate and banded. One of the two R2 chunks more rounded, the other subtriangular. R3 broadly concave, subhyaline (Fig. 10K, L).

**Female paratype.** Body length: 20.27; body width: 12.22; pronotum length × width: 9.34 × 4.95.

***Coloration*.** Terga yellowish brown to black (Fig. 10C). Vertex, eyes, post-clypeus and space between ocelli nearly black. Antennae yellowish brown. Ocelli and antennal sockets yellowish white. Ante-clypeus as well as upper and lower margins of labrum pale gray. Middle of labrum yellowish brown (Fig. 10H). Legs dark yellowish brown, tibia and spines dark yellowish brown to black. Sterna nearly black (Fig. 10D).

***Body*.** The widest point of pronotum near the hind margin, middle part with symmetrical black stripes, anterior whitish margin absent (Fig. 10F). Ocelli degraded to two white spots. Interocular space almost equal to the distance between antennal sockets, both bigger than the distance between ocelli (Fig. 10H). Front femur type C_1_. Arolia and pulvilli absent. Posterior margin of supra-anal plate slightly convex, emarginated medially. Cerci short and robust, not exceeding the posterior margin of supra-anal plate. Posterior margin of subgenital plate protruded and emarginated medially (Suppl. material 1: fig. S1C).

**Nymph.** Similar to the female, just a little paler in color.

**Ootheca.** Yellowish brown. Surface with densely parallel longitudinal lines. Ridges of serrated protuberances densely arranged with semicircular tips. No respiratory canals (Fig. 15B, K).

##### Etymology.

The species epithet is derived from the Latin words *longi* and *seta*, referring to the dense, long pubescence on the pronotum and head of the species.

##### Remark.

The genetic distance between this species and the remainder of the genus ranges from 9.18% to 18.74%, supporting it being a new species. The collection site of this species is close to the distribution site of *Epipolyphagawukong* Qiu, Che & Wang, 2019, and there may be a sympatric distribution between them.

#### ﻿ Pseudoeupolyphaga latizona

Taxon classificationAnimaliaBlattodeaCorydiidae

Han, Che & Wang sp. nov.

871A5165-EC7A-5754-B4EC-DB9D437B67BA

https://zoobank.org/6813330E-1E3B-4BBD-8189-DEBAA9C9FED4

**Figure 11.**
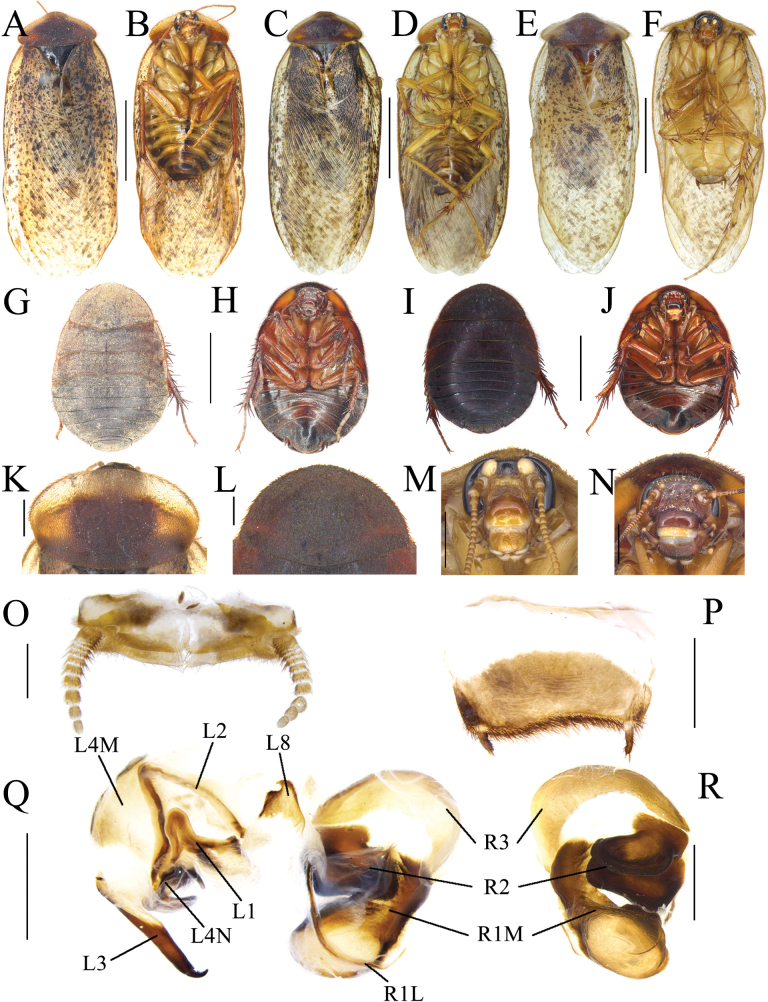
*Pseudoeupolyphagalatizona* Han, Che & Wang, sp. nov. **A, B, K, M, O–R** male holotype (Shimian County) **C–F** male paratype (Danba County) **G, H, L, N** female paratype (Shimian County) **I, J** female paratype (Danba County) **A** habitus, dorsal view **B** habitus, ventral view **C** habitus, dorsal view **D** habitus, ventral view **E** habitus, dorsal view **F** habitus, ventral view **G** habitus, dorsal view **H** habitus, ventral view **I** habitus, dorsal view **J** habitus, ventral view **K** pronotum, dorsal view **L** pronotum, dorsal view **M** head, ventral view **N** head, ventral view **O** supra-anal plate, ventral view **P** subgenital plate, ventral view **Q** genitalia, dorsal view **R** right phallomere, right-ventral view. Scale bars: 1.0 cm (**A–J**); 0.2 cm (**K–N**); 0.1 cm (**O–R**).

Figs 11A–R , 15C, D, L, M

##### Type material.

***Holotype***: China • male; Sichuan Province, Yaan City, Shimian County, Caoke Village; 20 Jul. 2022; Wei Han, Xin-Xing Luo leg. ***Paratypes***: China • 1 female, 5 nymphs & some oothecae, same collection data as holotype • 4 males; Sichuan Province, Ganzi Prefecture, Danba County; 12 Jul. 2017; Jian-Yue Qiu, Hao Xu leg • 1 male; Sichuan Province, Ganzi Prefecture, Danba County, Jiaju Zangzhai; 12 Jul. 2017; Hao Xu, Jian-Yue Qiu leg • 2 males; Sichuan Province, Ganzi Prefecture, Danba County, Zhanggu Town, Baiga Mountain; 14 Jun. 2013; Li He leg • 1 female, 3 nymphs, 3 oothecae; Sichuan Province, Ganzi Prefecture, Danba County, Zhanggu Town, Baiga Mountain; Oct. 2016; Jian-Yue Qiu leg • 1 female, 2 nymphs, 5 oothecae; Sichuan Province, Ganzi Prefecture, Danba County; 20 Feb. 2017; Jian-Yue Qiu leg • 1 male; Sichuan Province, Ganzi Prefecture, Danba County, Zhanggu Town, Baiga Mountain; 14 Jun. 2013; Li He leg • 4 nymphs, 1 ootheca; Sichuan Province, Ganzi Prefecture, Danba County, Zhanggu Town, Baiga Mountain; Feb. 2017; Lu Qiu leg.

##### Diagnosis.

The male of this species resembles the newly described species *P.baimaensis* sp. nov., but differs in having denser markings on the tegmina, darker abdominal coloration, and more distinct boundaries of yellow-black abdominal markings. The female of this species has slightly smaller ocelli compared to the latter. Additionally, the serrations of the ootheca of this species are very weak, whereas those of *P.baimaensis* sp. nov. are slightly stronger.

##### Description.

**Holotype. *Measurements* (mm).** Overall length (including tegmen): 33.70; body length: 20.94; body width (tegmina not included): 11.54; tegmen length × width: 29.29 × 9.91; pronotum length × width: 9.19 × 5.39.

***Coloration*.** Pronotum dark yellowish brown, covered with short yellowish setae. Anterior margin white (Fig. 11A, K). Maculae in tegmina and hind wings blackish brown (Fig. 11A, B). Eyes, vertex, and spaces between ocelli black. Ocelli and antennal sockets white. Post-clypeus dark yellowish brown. Ante-clypeus pale yellow. Base of labrum white, rest yellowish brown. Labial palpi and maxillary palpi yellowish brown, connections white (Fig. 11B, M). Legs yellowish brown, tibia and spines dark yellowish brown. Pulvilli and arolia white. Sterna yellow, margins, middle and distal part with black markings (Fig. 11B).

***Body*. *Head***: Sub-rounded, nearly completely hidden under pronotum. Eyes and ocelli developed. Ocelli ridge narrow, with a row of setae on the upper edge. A dimple present between the ocelli. Interocular space nearly equal to the distance between ocelli, and both narrower than the distance between antennal sockets. Clypeus developed (Fig. 11M). ***Pronotum***: Transverse oval, widest near the middle. Densely covered with setae on surface, with symmetrical stripes in the middle. Anterior whitish margin greatly broad, clearly delineated from the yellowish-brown areas (Fig. 11K). ***Tegmina and hind wings***: Densely covered with maculae, most of maculae small (Fig. 11A). ***Legs***: Slender, front femur type C_1_. Pulvilli and arolia present. ***Abdomen***: Supra-anal plate transverse, narrow and pubescent, posterior margin slightly protruded medially. Paraprocts simple. Hind margin of subgenital plate densely covered with setae, slightly asymmetrical, middle part slightly concave. Styli long (Fig. 11O, P). ***Genitalia***: The left protuberance of L1 robust, two posterior lobes and terminal protrusion strong. L2 arched. Genital hook (L3) straight, the hook small. L4M broad lamellate. Pda and paa developed, protrusions long. L8 irregular, subtriangular. R1M expanded terminally. R1L elongate, banded. R2 divided into two chunks. R3 broadly concave (Fig. 11Q, R).

**Female paratype (same locality as holotype).** Body length: 24.12 mm; body width: 16.33 mm; pronotum length × width: 11.81 × 6.85 mm.

***Coloration*.** Terga yellowish brown to blackish brown (Fig. 11G, I). Vertex and eyes black. Ocelli yellow. Antennae yellowish brown. Antennal sockets, base of labrum and two sides of ante-clypeus white. Middle of ante-clypeus yellow. Post-clypeus and middle of labrum reddish brown. Distal part of labrum black. Legs dark yellowish brown to reddish brown, spines reddish brown to black. Sterna reddish brown to black (Fig. 11H, J).

***Body*.** The widest point of pronotum near the hind margin, middle area with symmetrical dark stripe. Anterior whitish margin absent (Fig. 11L). Ocelli degraded to two spots. Interocular space almost equal to the distance between ocelli, both narrower than the distance between antennal sockets (Fig. 11N). Front femur type C_1_. Arolia and pulvilli absent. Posterior margin of supra-anal plate protruded, slightly emarginated medially. Cerci short and robust, not exceeding posterior margin of supra-anal plate. Posterior margin of subgenital plate protruding medially (Figs 11G–J, Suppl. material 1: fig. S1D).

**Nymph.** Similar to the female, a little paler in color.

**Ootheca.** Dark reddish brown to black. Surface with densely parallel longitudinal lines. Serrations of keel very weak. No respiratory canals (Fig. 15C, D, L, M).

##### Etymology.

The species epithet is derived from a combination of the Latin words *latus* and *zona*, which refers to the broad anterior whitish margin on the pronotum of the male.

##### Remark.

Samples from Danba County were previously identified as *P.yunnanensis* (Qiu et al. 2018). However, their tegmina maculae are significantly denser than those of *P.yunnanensis*. There are some differences between samples from Shimian County and Danba County: the former has a darker body coloration and dense but separate tegmina maculae, while the latter has a paler body coloration and with some fused maculae in tegmina. We also found a recently emerged male individual with dense but scattered forewing maculae and a yellowish-white abdomen (Fig. 11E, F). The genetic distance between the samples from Shimian County and Danba County of this species is 4.75%, leading to their designation as conspecific. Furthermore, the genetic distance between this species and members of the rest of the genus ranges from 12.92% to 20.90%, providing further support for its classification as a new species.

#### ﻿ Pseudoeupolyphaga baimaensis

Taxon classificationAnimaliaBlattodeaCorydiidae

Han, Che & Wang sp. nov.

D9130C9C-C7F6-58D3-BF72-9377DC1BDF54

https://zoobank.org/74F84569-2690-4C2A-A13F-64E02FEB3CB4

Figs 12A–L , 15E, N

##### Type material.

***Holotype***: China • male; Sichuan Province, Mianyang City, Pingwu County, Baima Village; 4 Aug. 2019; Lu Qiu leg. ***Paratype***: China • 1 female, same collection data as holotype.

##### Other material examined.

China • 10 oothecae; same collection data as holotype.

##### Diagnosis.

The male of this species resembles *P.latizona* sp. nov., but differs in having sparser markings on the tegmina, paler abdominal coloration, and less distinct boundaries of yellow-black abdominal markings. The female of this species has slightly larger ocelli compared to the latter. Additionally, the serrations of the ootheca of this species are slightly stronger than those of *P.latizona* sp. nov.

##### Description.

**Male holotype. *Measurements* (mm).** Overall length (including tegmen): 35.51; body length: 23.39; body width (tegmina not included): 11.53; tegmen length × width: 30.00 × 9.40; pronotum length × width: 10.28 × 5.91.

**Figure 12.**
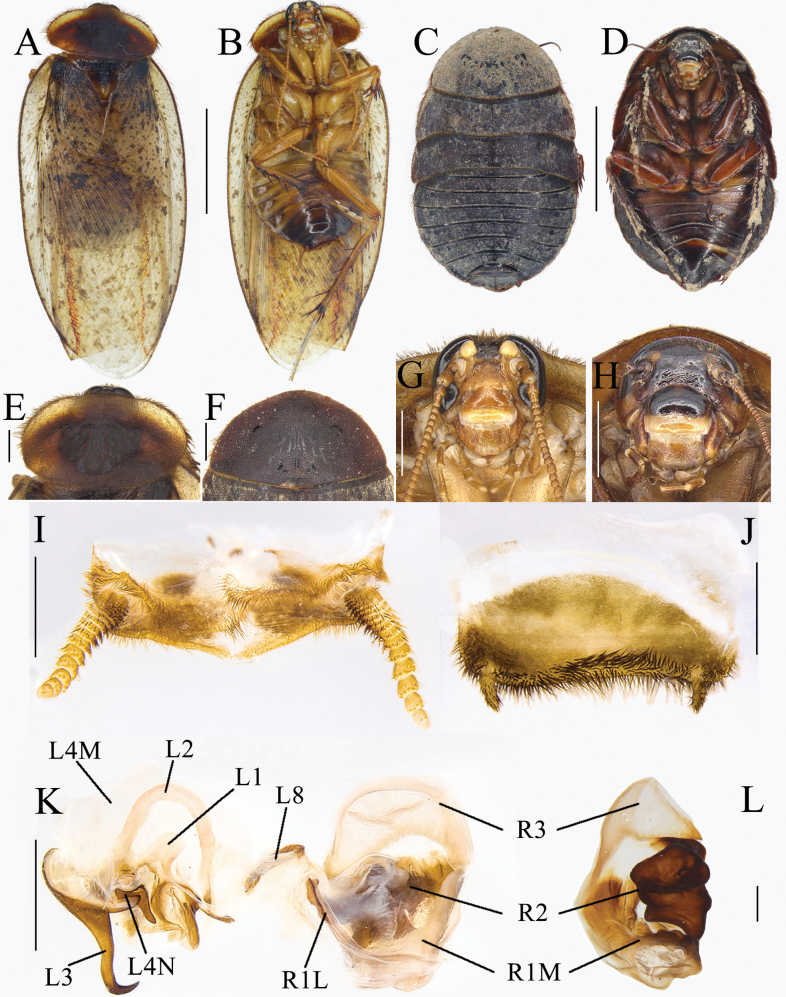
*Pseudoeupolyphagabaimaensis* Han, Che & Wang, sp. nov. **A, B, E, G, I–L** male holotype **C, D, F, H** female paratype. **A** habitus, dorsal view **B** habitus, ventral view **C** habitus, dorsal view **D** habitus, ventral view **E** pronotum, dorsal view **F** pronotum, dorsal view **G** head, ventral view **H** head, ventral view **I** supra-anal plate, ventral view **J** subgenital plate, ventral view **K** genitalia, dorsal view **L** right phallomere, right-ventral view. Scale bars: 1.0 cm (**A–D**); 0.2 cm (**E–H**); 0.1 cm (**I–L**).

***Coloration*.** Pronotum yellowish brown, anterior margin white. Tegmina and hind wings pale yellow, maculae blackish brown (Fig. 12A, E). Eyes, vertex, and spaces between ocelli black. Ocelli and ante-clypeus yellowish white. Antennal sockets white. Antennal sockets, post-clypeus, and labrum yellowish brown. Labial palpi and maxillary palpi yellowish brown, distal part and connections white (Fig. 12G). Legs yellowish brown, spines and outside of tibia dark yellowish brown to black. Pulvilli and arolia white. Sterna dark yellowish brown, margins and distal part black (Fig. 12B).

***Body*. *Head***: Sub-rounded, nearly completely hidden under pronotum. Eyes and ocelli developed. Ocelli ridge indistinct, with a row of setae on the upper edge. Interocular space nearly equal to the distance between ocelli, and both narrower than the distance between antennal sockets. Clypeus developed (Fig. 12G). ***Pronotum***: Transverse oval, widest near the middle. Densely covered with setae and pubescence, middle part with symmetrical stripe. Anterior whitish margin greatly broad and clearly delineated from yellowish brown areas (Fig. 12E). ***Tegmina and hind wings***: Densely covered with small maculae, maculae fused near the base (Fig. 12A). ***Legs***: Slender, front femur type C_1_. Pulvilli and arolia present (Fig. 12B). ***Abdomen***: Supra-anal plate transverse, narrow and pubescent, posterior margin slightly protruded medially. Paraprocts simple. Hind margin of subgenital plate flat, densely covered with setae. Styli columnar (Fig. 12I, J). ***Genitalia***: L1 weakly sclerotized, anterior protrusion round, the left protuberance robust, two posterior lobes curved. L2 arched, terminal round. Genital hook (L3) curved in the middle. L4M broad lamellate. Pda and paa developed, protrusions long. L8 long and narrow, flaky. R1M widely expanded at terminal part. R1L elongate, banded. R2 divided into two chunks. R3 broadly concave (Fig. 12K, L).

**Female paratype.** Body length: 25.19 mm; body width: 16.01 mm; pronotum length × width: 9.29 × 5.09 mm.

***Coloration*.** Terga blackish brown. Vertex, eyes, space between ocelli and post-clypeus black. Ocelli and antennae yellowish brown. Ante-clypeus and base of labrum yellowish white. Middle and distal part of labrum yellowish brown. Legs dark yellowish brown to black, spines black. Sterna nearly black, dark yellowish brown in most of central areas (Fig. 12C, D, H).

***Body*.** The widest point of pronotum near the hind margin, middle part with symmetrical black stripe. Anterior whitish margin absent (Fig. 12F). Ocelli degraded to two spots. Interocular space almost equal to the distance between ocelli, both narrower than the distance between antennal sockets (Fig. 8H). Front femur type C_1_. Arolia and pulvilli absent. Posterior margin of supra-anal plate protruded, emarginated medially. Cerci short, not exceeding posterior margin of supra-anal plate. Posterior margin of subgenital plate protruded, emarginated medially (Figs 12C, D, Suppl. material 1: fig. S1E).

**Nymph.** Unknown.

**Ootheca.** Yellowish brown. Surface with parallel longitudinal lines. Serrations of keel weak, terminal blunt. No respiratory canals (Fig. 15E, N).

##### Etymology.

The species epithet is derived from the type locality, Baima Village, in Pingwu County, Mianyang City, Sichuan Province.

##### Remark.

The genetic distance between this species and the remaining members of the genus ranges from 12.92% to 19.70%, providing support for its classification as a new species.

#### ﻿ Pseudoeupolyphaga pilosa

Taxon classificationAnimaliaBlattodeaCorydiidae

(Qiu, Che & Wang, 2018)

097BDB37-4663-56DA-8E0D-859D7FBEE141

**Figure 13.**
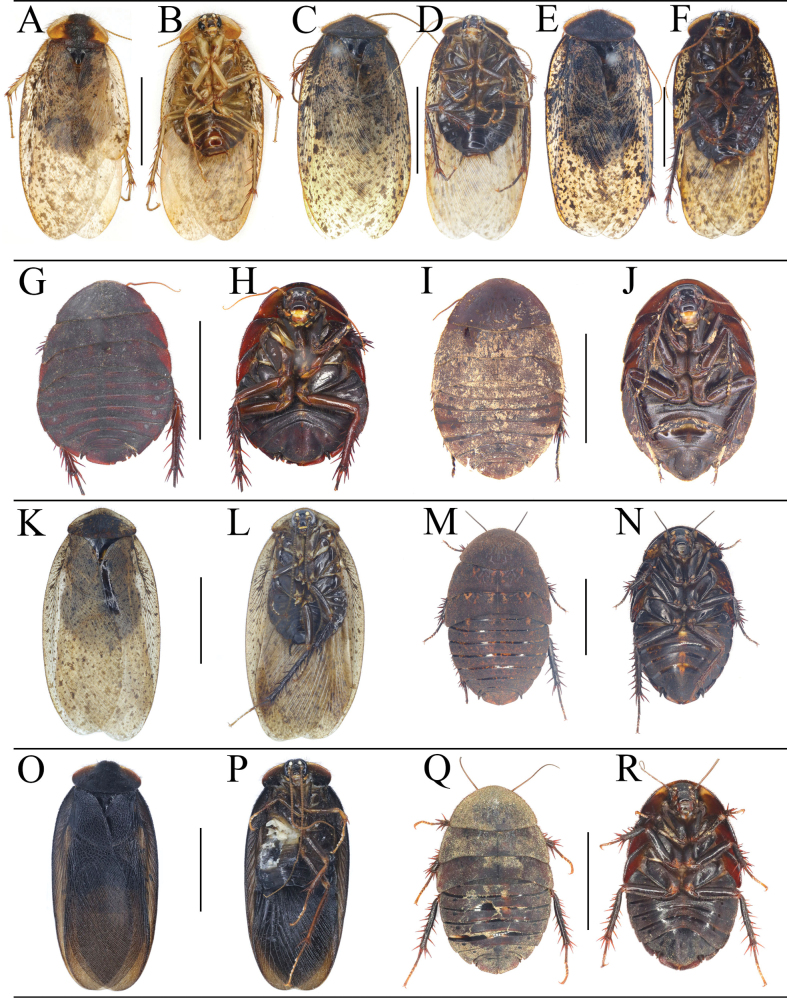
*Pseudoeupolyphagapilosa* (Qiu, Che & Wang, 2018) (**A–J)***Pseudoeupolyphagafengifengi* (Qiu, Che & Wang, 2018) (**K–N)***Pseudoeupolyphagafusca* (Chopard, 1929) (**O–R**) **A, B** male of Luodatang countryside, **C, D** male of Yulong Snow Mountain, **E, F** male of Wenbi Mountain, **G, H** female of Pantiange Township, **I, J** female of Yulong Snow Mountain, **K, L** male of Dahei Mountain, **M, N** female of Dahei Mountain, **O, P** male of Cang Mountain, **Q, R** female of Cang Mountain. **A, C, E, G, I, K, M, O, Q** habitus, dorsal view **B, D, F, H, J, L, N, P, R** habitus, ventral view. Scale bars: 1.0 cm (**A–R**).

Figs 13A–J , 15F, G, O, P

Eupolyphaga pilosa Qiu, Che & Wang, 2018: 50; Qiu et al. 2019: 11 (catalogue).
Pseudoeupolyphaga pilosa : Han et al. 2024: 166.

##### Type locality.

“Yunnan Province, Diqing Prefecture, Weixi County, Pantiange Township, A valley Near Zhazi; 2970 m”

##### New material examined.

China • 1 male, 2 females; Yunnan Province, Lijiang City, Yulong Snow Mountain, Blue Moon Valley; 24 Jul. 2022; Wei Han, Lin Guo leg • 1 male, 1 female; Yunnan Province, Lijiang City, Wenbi Mountain; 24 Jul. 2022; Wei Han, Lin Guo leg • 1 male, 4 nymphs; Yunnan Province, Weixi County, Badi Village, Luodatang countryside; 25 Jul. 2022; Wei Han, Xin-Xing Luo, Lin Guo leg.

##### Remarks.

This species was previously only documented in Pantiange Township, Weixi County, Yunnan Province. However, a recent collection in Yunnan has expanded its distribution range. In samples collected at various sites, the density of markings on the male tegmina varied (Fig. 13A–F). Markedly sparser markings were observed on the samples from Pantiange Township (Qiu et al. 2018: fig. 8A, B) and Luodatang countryside in Weixi County (Fig. 13A, B) compared to those from Yulong Snow Mountain (Fig. 13C, D) and Wenbi Mountain (Fig. 13E, F). Additionally, the male abdomens of samples from Pantiange Township, Yulong Snow Mountain, and Wenbi Mountain were dark brown to black, while those from Luodatang countryside were yellowish-brown.

Genetic distance analyses revealed that the genetic distance between samples from the four different collection sites ranged from 3.92% to 7.54%. Given the proximity of these new distributions to the type locality, and the absence of significant differences in oothecae (Fig. 15F, G, O, P; Qiu et al. 2018: fig. 38I, Q), the samples from the new location were temporarily classified as distinct geographic populations of *P.pilosa*.

#### ﻿ Pseudoeupolyphaga fengi fengi

Taxon classificationAnimaliaBlattodeaCorydiidae

(Qiu, Che & Wang, 2018)

C30EDFD5-9459-5CBA-BCDC-02C4670FB084

Figs 13K–N , 15H, Q

Eupolyphaga fengi fengi Qiu, Che & Wang, 2018: 42; Qiu et al. 2019: 11 (checklist).
Pseudoeupolyphaga fengi fengi : Han et al. 2024: 166.

##### Type locality.

“Yunnan Province, Chuxiong City, Zixi Mountain; 2397 m”

##### New material examined.

China • 1 male, 2 females & 1 ootheca; Sichuan Province, Panzhihua City, Dahei Mountain, Xiaoshilin Pass; 22 Jul. 2022; Wei Han, Lin Guo leg.

##### Ootheca.

Light reddish brown. Longitudinal lines densely arranged. Serrated protuberances sparsely arranged, tips subtriangular and slightly tilted. No respiratory ducts (Fig. 13H, Q).

##### Remarks.

The male specimen from Zixi Mountain has pale yellowish-brown tegmina, a dark brown abdomen, and legs with yellow markings (Qiu et al. 2018: fig. 10E, F). While male samples from Dahei Mountain display pale grayish-brown tegmina, a blackish brown abdomen and legs, and yellow markings on the abdomen (Fig. 13K, L). The density of markings on tegmina is nearly identical in both location samples. Genetic distances range from 0% to 0.8% between the samples from Zixi Mountain, and from 6.6% to 7.1% between the samples from Zixi Mountain and Dahei Mountain. Since the genetic distances between the samples from Zixi Mountain and Dahei Mountain did not significantly differ, and the distribution of tegmina markings as well as the degree of density were almost identical, the differences in coloration and markings between the samples from Dahei Mountain and those from the type locality, Zixi Mountain, are temporarily considered to be intraspecific variation.

#### ﻿ Pseudoeupolyphaga fusca

Taxon classificationAnimaliaBlattodeaCorydiidae

(Chopard, 1929)

BF867D0D-E893-5539-93DC-2A23B7EC3087

Figs 13O–R , 15I, R

Eupolyphaga fusca Chopard, 1929: 270; Wu, 1935: 29; Princis, 1952: 35; Bey-Bienko, 1957: 896; Princis, 1962: 55; Qiu et al. 2018: 28; Qiu et al. 2019: 11 (catalogue).
Pseudoeupolyphaga fusca : Han et al. 2024: 166.

##### Type locality.

“Yunnan Province, Kunming City”

##### New material examined.

China • 1 male, 2 females & 5 oothecae; Yunnan Province, Dali City, Cangshan National Geopark, Yudai Road; 29 Jul. 2022; Wei Han, Xin-Xing Luo leg.

##### Ootheca.

Light yellowish brown. Longitudinal lines densely arranged but not prominent. Serrated protuberances sparsely arranged with subtriangular tips. No respiratory ducts (Fig. 15I, R).

##### Remarks.

This species is the only one in the genus with unicolored tegmina; the remaining species have spotted tegmina. It has the smallest interspecific genetic distance (6.61%) with *P.pilosa* in the genus. However, it can be distinguished from the latter based on tegmina coloration alone.

#### ﻿ Pseudoeupolyphaga simila

Taxon classificationAnimaliaBlattodeaCorydiidae

(Qiu, 2022)

D5F8DC05-26B1-5551-A938-F8C0268D0DF4

Fig. 14A–J

Eupolyphaga simila Qiu, 2022 in Han et al. 2022: 81.
Pseudoeupolyphaga simila : Han et al. 2024: 166.

##### Type locality.

“Sichuan Province, Lixian County, Miyaluo Town, Siboguo Village; 2944 m”.

##### New material examined.

China • 3 males, 1 nymph; Sichuan Province, Aba Prefecture, Li County, Parktou Township, Tazigou; 22 Apr. 2023; Wei Han leg • 1 male, 1 female; Sichuan Province, Aba Prefecture, Li County, Dagou Village; 18 Apr. 2023; Wei Han leg.

##### Remarks.

**Figure 14.**
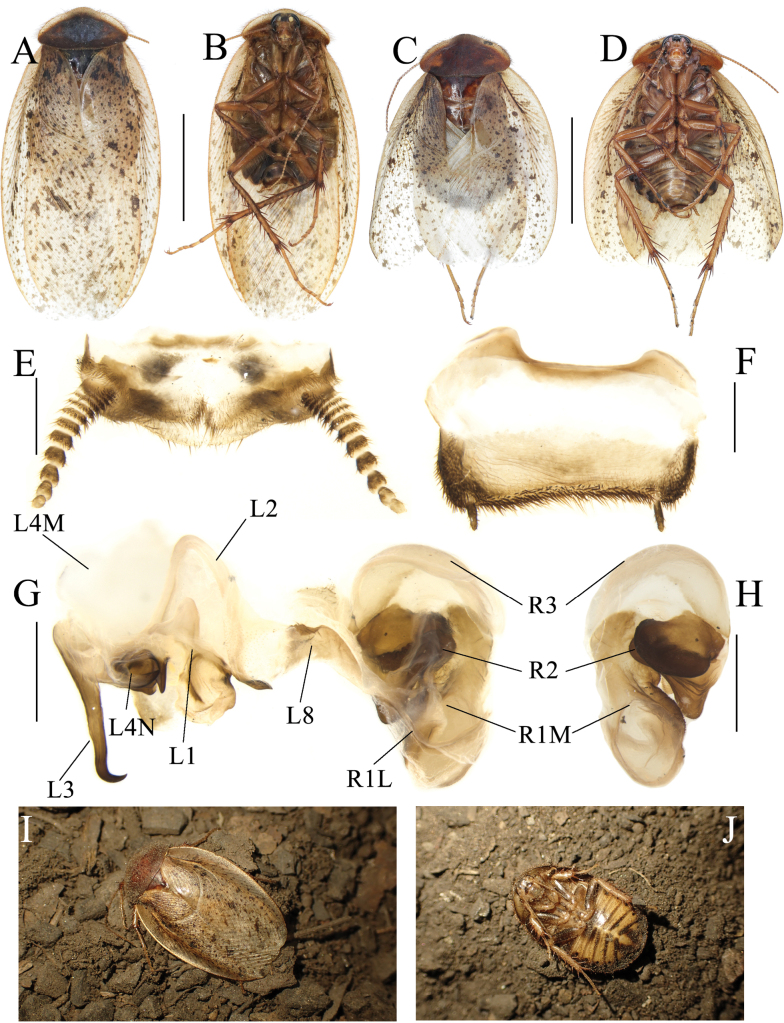
*Pseudoeupolyphagasimila* (Qiu, 2022) **A, B** male from Dagou Village, Li County, Aba Prefecture, Sichuan Province **C–J** male and nymph from Tazigou, Putou Township, Li County, Aba Prefecture, Sichuan Province **A** habitus, dorsal view **B** habitus, ventral view **C** habitus, dorsal view **D** habitus, ventral view **E** supra-anal plate, ventral view **F** subgenital plate, ventral view **G** genitalia, dorsal view **H** right phallomere, right-ventral view **I** a living male, dorsal view **J** a living nymph, ventral view. Scale bars: 1.0 cm (**A–D**); 0.1 cm (**E–H**).

There was almost no difference in the external morphology between samples from Dagou Village (Fig. 14A, B) and the type locality Sibogo Village (Han et al. 2022: fig. 6A, B), aside from slightly denser tegmina markings in the former. The most discernible difference between Tazigou samples (Fig. 14C, D) and those from Sibogo Village was the relatively shorter and broader tegmina. Measurements for the Tazigou samples were as follows (mm): overall length: 21.87, body length: 16.71, body width (tegmina not included): 9.89, tegmen length × width: 18.44 × 8.13, and pronotum length × width: 8.37 × 3.88. Regarding genetic distance, it was 3.52% between Sibogo Village and Dagou village, 5.31% between Sibogo Village and Tazigou samples, and 5.12% between Dagou Village and Tazigou samples. Geographically, none of the three regions are more than thirty kilometers apart from each other. Hence, samples from both Dagou village and Tazigou are classified as *P.simila*.

**Figure 15.**
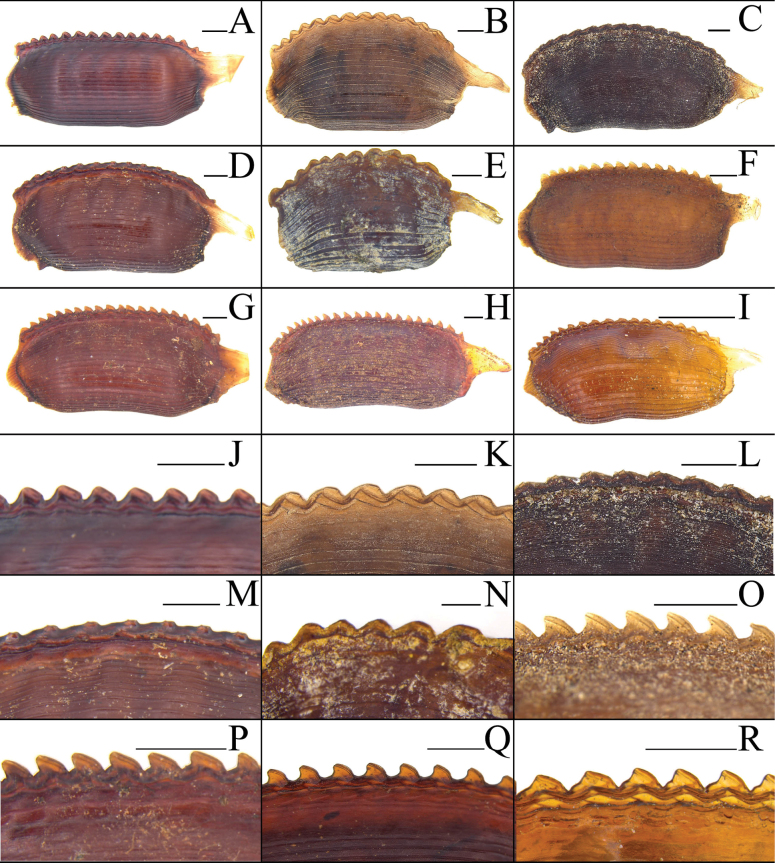
Oothecae of *Pseudoeupolyphaga*, lateral view (**A–I**) and close-up view to show the serrations (**J–R**) **A, J***P.deficiens* sp. nov. **B, K***P.longiseta* sp. nov. **C, D, L, M***P.latizona* sp. nov. **C, L** from Caoke Village **D, M** from Danba County **E, N***P.baimaensis* sp. nov. **F, G, O, P***P.pilosa* (Qiu, Che & Wang, 2018) **F, O** from Yulong Snow Mountain **G, P** from Wenbi Mountain **H, Q***P.fengifengi* (Qiu, Che & Wang, 2018) from Dahei Mountain **I, R***P.fusca* (Chopard, 1929) from Cang Mountain. Scale bars: 0.1 cm (**A–R**).

## ﻿Discussion

Incorporating molecular data could provide more reliable evidence for species identification within Corydioidea (Trotter et al. 2017; Han et al. 2022). Our results show that all morphologically identified species are supported by molecular data. However, the boundaries between interspecific and intraspecific genetic distances in *Pseudoeupolyphaga* remain unclear. The maximum intraspecific genetic distance within the genus is 7.54% (*P.pilosa*, samples from Luodatang countryside and Wenbi Mountain), while the minimum interspecific genetic distance is 6.61% (*P.pilosa* and *P.fusca*), resulting in overlapping intraspecific and interspecific genetic distances for the COI marker. This situation is detrimental to the delimitation of some morphologically similar specimens. Some studies have pointed out that the species’ limited migratory capacity and substantial geographic isolation of their ranges may account for the larger intraspecific genetic distances (Qiu et al. 2018; Han et al. 2022). Thus, the delimitation of species in the genus should consider not only morphological and molecular differences, but also differences in geographic distance. In the future, broader sampling, more comprehensive genetic data collection, and consideration of geographic distribution or chromosome number, coupled with meticulous analyses, could facilitate comprehension of the species formation and ultimately improve species delimitation efforts.

The structure of female genitalia can serve as useful characters for species identification in Blattodea, albeit with variations in the sclerites utilized. For instance, key characters include the basivalvula, laterosternal shelf, and spermatheca in *Cryptocercus* Scudder, 1862 (Wang et al. 2015; Bai et al. 2018); and the anterior arch and basivalvula in *Anaplecta* Burmeister, 1838 (Zhu et al. 2022). Previous investigations of female genitalia within Corydioidea were limited, with descriptions available for only eight species across four genera (McKittrick 1964; Mackerras 1968; Grandcolas 1993).

In this study, we conducted a comparative analysis of the female external genitalia and spermathecae among eight species of *Eupolyphaga* and 15 species and subspecies of *Pseudoeupolyphaga*. Our findings revealed consistent structural compositions among these species, with variations observed in the degree of sclerotization in some sclerites. However, in both genera, the roles of the female external genital sclerites and spermathecae in species delimitation are not the same. Among these genital structures, the spermatheca, spermathecal plate, and basivalvula exhibited the most significant interspecific variation in *Eupolyphaga*. They can be used as reliable characters for female identification of this genus, alone or in combination. The morphology of spermatheca in *Eupolyphaga* species exhibits variability, and the females of the eight species can be distinguished based on the number, morphology, and mode of ampulla attachment (Fig. 5I–P). Additionally, the shape of the spermathecal plate varies significantly in *E.bicolor* sp. nov., *E.nigra* sp. nov., *E.hupingensis*, *E.robusta*, and *E.hanae* (Fig. 5A, B, D, G, H), distinguishing each species from the others. Furthermore, the basivalvula also serves as a distinguishing feature for species identification. For instance, in *E.hupingensis* (Fig. 5D), its shape differs distinctly from that of other species, while in *E.hanae* (Fig. 5H), the anterior margins of the two lobes are notably toothed and prominent. While most sclerites of the female genitalia in *Pseudoeupolyphaga* are poorly sclerotized and lack distinct boundaries, the other well-sclerotized sclerites (spermathecal plate and basivalvula) are almost identical in shape, as are the spermathecae. This makes them well suited as synapomorphy of the genus, but not effective for species delimitation. In the future, the study of female genitalia in more genera should be considered to reveal more about their taxonomic significance and their evolutionary patterns.

## Supplementary Material

XML Treatment for Eupolyphaga

XML Treatment for Eupolyphaga bicolor

XML Treatment for Eupolyphaga nigra

XML Treatment for Eupolyphaga udenostyla

XML Treatment for Eupolyphaga hupingensis

XML Treatment for Eupolyphaga miracidia

XML Treatment for Eupolyphaga sinensis

XML Treatment for Eupolyphaga robusta

XML Treatment for Eupolyphaga hanae

XML Treatment for Pseudoeupolyphaga

XML Treatment for Pseudoeupolyphaga flava

XML Treatment for Pseudoeupolyphaga deficiens

XML Treatment for Pseudoeupolyphaga magna

XML Treatment for Pseudoeupolyphaga longiseta

XML Treatment for Pseudoeupolyphaga latizona

XML Treatment for Pseudoeupolyphaga baimaensis

XML Treatment for Pseudoeupolyphaga pilosa

XML Treatment for Pseudoeupolyphaga fengi fengi

XML Treatment for Pseudoeupolyphaga fusca

XML Treatment for Pseudoeupolyphaga simila

## ﻿Additional information

### Author ORCIDs

Wei Han https://orcid.org/0000-0002-7243-1657

Zong-Qing Wang https://orcid.org/0000-0001-9413-1105

### Data availability

All of the data that support the findings of this study are available in the main text or Supplementary Information.

## Supplementary materials

Supplementary material 1 Female external genitalia and spermatheca of 15 species and subspecies in *Pseudoeupolyphaga* Data type jpg https://binary.pensoft.net/file/1128152This dataset is made available under the Open Database License (http://opendatacommons.org/licenses/odbl/1.0/). The Open Database License (ODbL) is a license agreement intended to allow users to freely share, modify, and use this Dataset while maintaining this same freedom for others, provided that the original source and author(s) are credited.Wei Han, Yan-Li Che, Pei-Jun Zhang, Zong-Qing Wang

Supplementary material 2 Collecting information for the samples included in Fig. 6 and fig. S1 Data type xls https://binary.pensoft.net/file/1128153This dataset is made available under the Open Database License (http://opendatacommons.org/licenses/odbl/1.0/). The Open Database License (ODbL) is a license agreement intended to allow users to freely share, modify, and use this Dataset while maintaining this same freedom for others, provided that the original source and author(s) are credited.Wei Han, Yan-Li Che, Pei-Jun Zhang, Zong-Qing Wang

Supplementary material 3 Genetic distances between COI genes of *Eupolyphaga* and *Pseudoeupolyphaga* species used in Fig. 1 Data type xls https://binary.pensoft.net/file/1128154This dataset is made available under the Open Database License (http://opendatacommons.org/licenses/odbl/1.0/). The Open Database License (ODbL) is a license agreement intended to allow users to freely share, modify, and use this Dataset while maintaining this same freedom for others, provided that the original source and author(s) are credited.Wei Han, Yan-Li Che, Pei-Jun Zhang, Zong-Qing Wang

Supplementary material 4 Molecular data matrix (COI genes) used in this study Data type fas https://binary.pensoft.net/file/1128155This dataset is made available under the Open Database License (http://opendatacommons.org/licenses/odbl/1.0/). The Open Database License (ODbL) is a license agreement intended to allow users to freely share, modify, and use this Dataset while maintaining this same freedom for others, provided that the original source and author(s) are credited.Wei Han, Yan-Li Che, Pei-Jun Zhang, Zong-Qing Wang
