# Supplementary figures and images for: ﻿New species of Eupolyphaga Chopard, 1929 and Pseudoeupolyphaga Qiu & Che, 2024 (Blattodea, Corydioidea, Corydiinae), with notes on their female genitalia
Source: Zookeys. 2024 Sep 4;1211:151–91. doi: 10.3897/zookeys.1211.128805 (PMC11391126; doi:10.3897/zookeys.1211.128805)

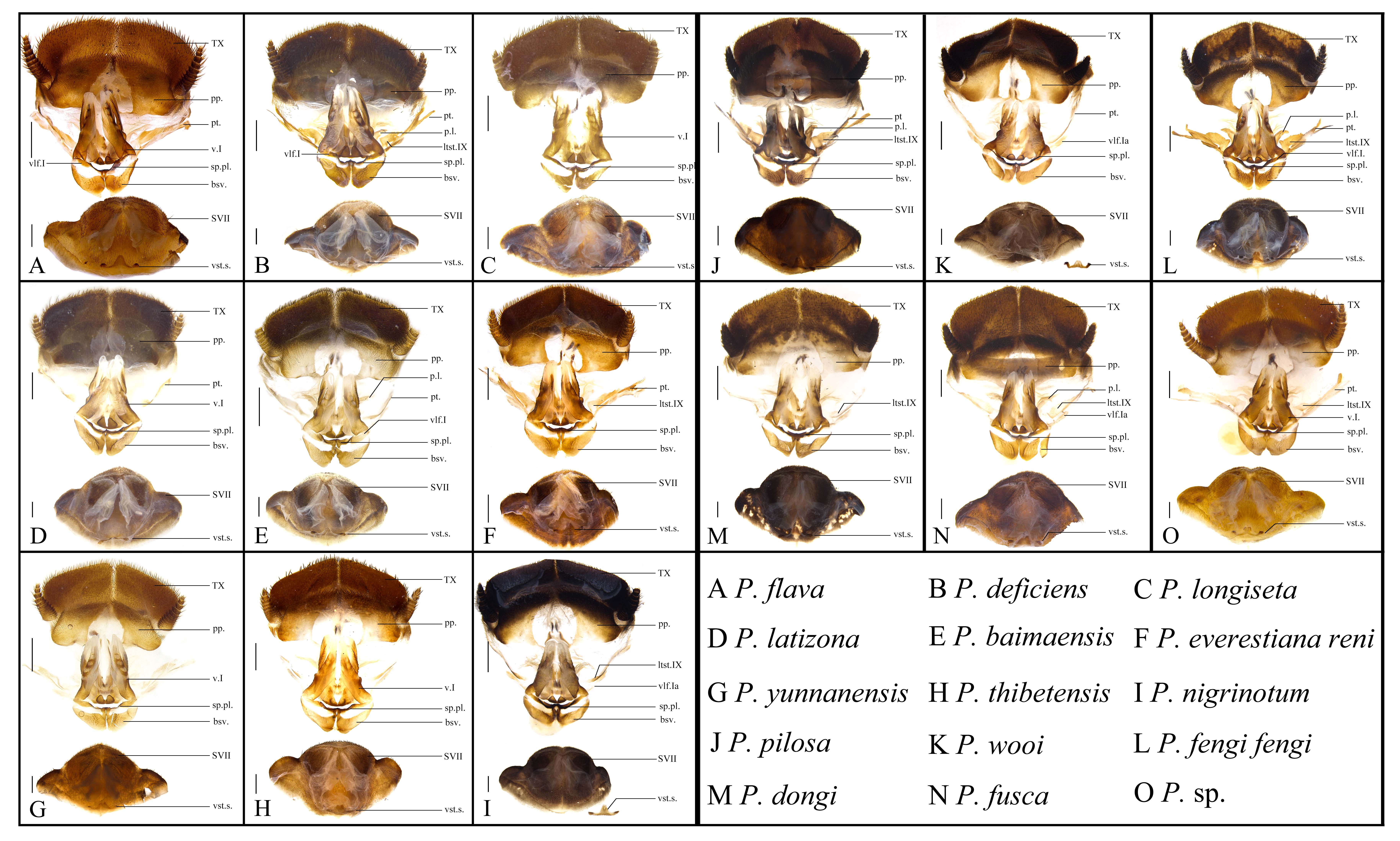

Supplement: Supplementary material 1 — Female external genitalia and spermatheca of 15 species and subspecies in Pseudoeupolyphaga [file zookeys-1211-151_article-128805__-s001.jpg]
